# Active Ingredients from Chinese Medicine for Combination Cancer Therapy

**DOI:** 10.7150/ijbs.77720

**Published:** 2023-07-09

**Authors:** Xuan Wang, Jing Li, Ruie Chen, Ting Li, Meiwan Chen

**Affiliations:** 1 State Key Laboratory of Quality Research in Chinese Medicine, Institute of Chinese Medical Sciences, University of Macau, Macau, 999078, China.; 2 State Key Laboratory of Quality Research in Chinese Medicines, Macau Institute for Applied Research in Medicine and Health, Macau University of Science and Technology, Macau, 999078, China.; 3 MoE Frontiers Science Center for Precision Oncology, University of Macau, Macau, 999078, China.

**Keywords:** Chinese medicine, Active ingredients, Combination cancer therapy, Nanomedicine

## Abstract

Combination therapy against cancer has gained increasing attention because it can help to target multiple pathways to tackle oncologic progression and improve the limited antitumor effect of single-agent therapy. Chinese medicine has been studied extensively in cancer therapy and proven to be efficacious in many cases due to its wide spectrum of anticancer activities. In this review, we aim to summarize the recent progress of active ingredients from Chinese medicine (AIFCM) in combination with various cancer therapeutic modalities, including chemotherapy, gene therapy, radiotherapy, phototherapy and immunotherapy. In addition to highlighting the potential contribution of AIFCM in combination cancer therapy, we also elucidate the underlying mechanisms behind their synergistic effect and improved anticancer efficacy, thereby encouraging the inclusion of these AIFCM as part of effective armamentarium in fighting intractable cancers. Finally, we present the challenges and future perspectives of AIFCM combination therapy as a feasible and promising strategy for the optimization of cancer treatment and better clinical outcomes.

## 1. Introduction

Cancer is still a major threat to public health and one of the leading causes of mortality worldwide 1. Extensive efforts have been made to develop various cancer treatment modalities, including surgery, chemotherapy, gene therapy, radiotherapy, phototherapy, and immunotherapy. However, owing to the physiological heterogeneity of cancer and certain obstacles such as the drug resistance of therapeutic agents, limited tumoricidal penetration depth of phototherapy, radioresistance and immune tolerance, these monotherapies often exhibit limited therapeutic outcomes and have inevitable side effects or adverse reactions to cancer patients in clinical practice 2. To address these challenges, a variety of anticancer strategies have been developed. Combination therapy, which makes use of different therapeutic modalities, is regarded as one of the most promising options among these treatments. It can inhibit cancer through complementary mechanisms and yield favorable therapeutic outcomes, such as enhanced tumor growth inhibition, reduced adverse effects, drug resensitization, lowered relapse rate and metastasis inhibition 3, 4. Therefore, a better understanding of the mechanisms underlying of different combination therapy strategies would be meaningful for cancer treatment 5.

Chinese medicine, an important component of complementary and alternative medicine in the world, is supported by rich clinical experience gleaned over thousands of years of practice process in China and other Asian countries 6. Modern clinical studies have demonstrated that Chinese medicine could prolong survival and improve the quality of life for patients undergoing treatment of various cancer such as liver cancer, gastric cancer, and lung cancer 7, 8. Recently, a series of pharmacological studies have shown that active ingredients from Chinese medicine (AIFCM) could effectively induce tumor inhibition, increase the sensitivity of tumors to other therapies, improve immune function, and inhibit angiogenesis and metastasis, which may exert prominent synergistic therapeutic effect in cancer combination therapy 9, 10. With the development of Chinese medicine chemistry, different kinds of AIFCM have been isolated and identified, showing the advantages of multi-channel and multi-target in cancer treatment. Moreover, it should be emphasized that various AIFCM exhibit broad-spectrum anticancer activities 11. For example, several polyphenols, such as curcumin, quercetin, and resveratrol, can target multiple pathways related to tumor proliferation and increase the sensitivity of cancer cells in chemotherapy and radiotherapy 12, 13.

Due to these incredible characteristics, AIFCM have been considered as great candidates in combination therapy with other therapeutic modalities for synergistically combating cancer to achieve more effective therapeutic outcomes than mono-therapy approach 12. Moreover, with the development of nanotechnology, various nanocarriers have been used to facilitate the delivery of AIFCM and combination drugs to the tumor site, providing further opportunities for the application of AIFCM in cancer therapy. In particular, some polyphenols such as (-)-epigallocatechin-3-O-gallate (EGCG) in AIFCM could involve in the construction of nanomaterials with their polyphenol structures, and act as nanocarriers for the delivery of drugs, proteins, genes for cancer therapy 14-16. Herein, our review summarizes the recent studies on combination therapy of AIFCM and conventional tumor therapy, including chemotherapy, gene therapy, phototherapy, radiotherapy and immunotherapy. The diverse therapeutic effects and the underlying mechanisms of AIFCM in combination therapy are highlighted, together with a brief discussion on the advantages, obstacles and clinical application potential of AIFCM-based combination therapy. The remarkable roles of AIFCM in combined anticancer therapy pave the way for applying AIFCM for clinical cancer treatment.

## 2. Combination Modalities

### 2.1 AIFCM Combined with Chemotherapy

Chemotherapy is a major approach in the clinic for inhibiting primary tumors and tumor metastasis whereby rapidly proliferating tumor cells are killed by toxic chemical compounds 17. At present, platinum-based drugs including cisplatin, carboplatin; and oxaliplatin are still used for the first-line chemotherapy in cancer treatment. However, chemotherapeutic drugs are often accompanied by side effects that are of great concern, such as ototoxicity, nephrotoxicity and neurotoxicity 18. In addition, some patients have innated or acquired resistance to chemotherapeutic drugs, resulting in low therapeutic effect, tumor metastasis and tumor recurrence 19. Besides, owing to the heterogeneity of tumor tissue, tumor cells with variable genotypes and phenotypes show different degrees of sensitivity to chemotherapeutic drugs 20. Moreover, tumor metastasis inhibition by chemotherapy is rarely satisfactory because of the complexity of biological processes in tumor progression. In consequence, a single chemotherapeutic drug cannot eradicate cancer cells completely sometimes, and the surviving cancer cells can lead to tumor recurrence 21. Therefore, new combination therapies are needed to improve the clinical efficacy of current chemotherapy and reduce the related side effects.

**Figure 1:**
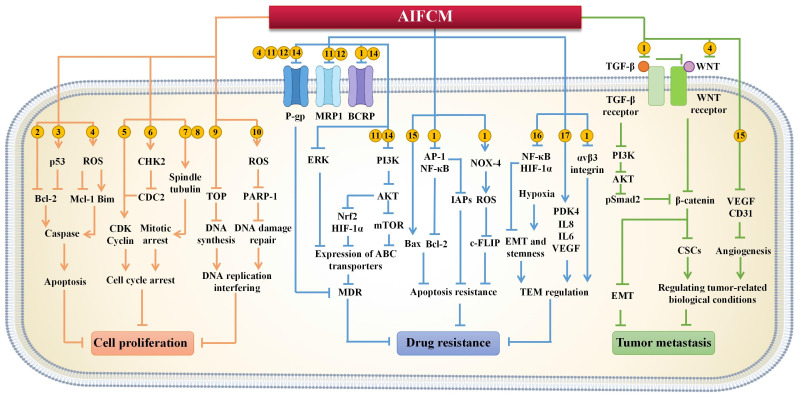
The mechanism of active ingredients from Chinese medicine (AIFCM) in chemotherapy enhancement by inhibiting tumor proliferation, reversing multi-drug resistance and preventing cancer metastasis. Active ingredients from Chinese medicine: 1. Curcumin; 2. Genistein; 3. Baicalein; 4. Tetrandrine; 5. Triptolide; 6. β-elemene; 7. Paclitaxel; 8. Vincristine; 9. Camptothecin; 10. Arsenic trioxide; 11. Matrine; 12. Resveratrol; 13. Glaucine; 14. Nuciferine; 15. Andrographolide; 16. Ginsenoside; 17. Quercetin.

In various types of cancer treatment clinical trials, AIFCM or its herbal preparations have been used for adjuvant chemotherapy to reduce side effects and complications of chemotherapy (such as pain, myelosuppression and gastrointestinal side effects, etc.), sensitize tumor cells to chemotherapy, and enhance the therapeutic efficacy 22, 23. For example, in the adjuvant chemotherapy of vinorelbine plus cisplatin/carboplatin for treating patients with non-small cell lung cancer, the treatment of different formulations containing Chinese medicine such as *Radix astragalus*, *Radix adenophorae*, and *Radix ophiopogonis*, alleviated the side effects of patients including fatigue, pain, diarrhea, vomiting and other adverse symptoms 6, 24. Compared with single-agent therapy, AIFCM-assisted combination chemotherapy can produce synergistic effects in the form of multi-target and multi-pathway, and improve anti-tumor efficacy. A variety of AIFCM such as baicalein, rhein, and β-elemene have been proved to significantly improve the efficacy of chemotherapy by inhibiting tumor proliferation, reversing multi-drug resistance and preventing cancer metastasis 25. Therefore, the strategy of AIFCM combined with chemotherapy will solve some of the problems existing in chemotherapy and promote the therapeutic effect, which shows a great prospect in cancer therapy (Figure 1).

#### 2.1.1 Inhibition of Tumor Proliferation

Sustained proliferation and resistance to cell death are two hallmarks in the development of cancer. Considering these properties, conventional chemotherapeutic agents can inhibit fast-growing tumor cells by interfering with cell division. However, a single chemotherapeutic drug usually fails to circumvent the physiological complexity of tumors, which leads to unsuccessful tumor therapy 26. AIFCM (such as usnic acid, curcumin, et al.) can inhibit tumor proliferation and enhance the efficacy of chemotherapy by inducing apoptosis, blocking the cell cycle of cancer cells, and interfering with DNA replication 27, 28. Thus, AIFCM could be applied in combinational chemotherapy to improve the therapeutic effect.

Most chemotherapeutic drugs exert their antitumor effect by inducing cell apoptosis, which is mediated by caspases in an extrinsic pathway or intrinsic pathway and leads to a series of biological and morphological changes, such as cleavage of poly (ADP-ribose) polymerase (PARP) and DNA fragmentation 29. In the intrinsic apoptotic pathway, the B-cell lymphoma-2 (Bcl-2) family proteins, which could be classified into anti-apoptotic proteins (e.g., Bcl-2, Bcl-xL, Mcl-1) and pro-apoptotic proteins (e.g., Bax, Bak, Bim), are considered as key regulators responsible for the fate of cell survival or death 30. Specifically, the pro-apoptotic proteins can activate the downstream caspase pathway and induce mitochondria dysfunction for promoting apoptosis of tumor cells, while the anti-apoptotic proteins may show the opposite effect. This suggests that the balance of pro-/anti-apoptotic proteins of Bcl-2 family is meaningful for cancer treatment. Interestingly, AIFCM was found to be involved in the balance of pro/anti-apoptotic protein for promoting the up-regulation of caspases in cancer cells, thus synergistically inducing apoptosis with chemotherapeutic drugs and ultimately improving anti-cancer efficacy. For examples, genistein, which is a soybean isoflavone existing in Chinese herbal medicine such as *Pueraria lobata*, *Vietnamese Sophora* root and *Net Cliffbean*, combined with arsenic trioxide to significantly shift the balance of Bax/Bcl-2 proteins and thus increased the expression of caspase-3 and caspase-9, leading to efficient tumor inhibition in hepatocellular carcinoma (HCC). And compared with using arsenic trioxidesize or genistein alone, the combination therapy suppressed tumor growth and reduced the proliferation index in HepG2 tumor-bearing mice more efficiently 31. Besides, AIFCM can up-regulate tumor suppressor genes, such as p53, to transcriptionally activate pro-apoptotic proteins and decrease anti-apoptotic proteins in Bcl-2 family for synergistic apoptosis induction 32. Baicalein is a flavone derived from the roots of *Scutellaria baicalensis*. It was found to increase the expression of p53 by 5-fold compared with the treatment of 10-hydroxycamptothec (HCPT) alone in human gastric carcinoma cells when combined with HCPT. The combinational drugs synergistically caused a significant imbalance in Bax/Bcl-2, and led to increased levels of caspase-3, caspase-9 and PARP-1, ultimately resulting in potent tumor suppression in BGC823 cell-xenografted mice 33. In addition, AIFCM were reported to generate excessive reactive oxygen species (ROS) to promote cell apoptosis in chemotherapy. In specific, ROS are a class of highly reactive molecules derived from oxygen (O_2_), such as hydroxyl radical (HO•), hydrogen peroxide (H_2_O_2_) and superoxide anion (O^2-^). Excessive ROS caused by AIFCM could cause oxidative stress and mitochondrial dysfunction in cancer cells, which would contribute to the regulation of Bcl-2 family proteins and finally the caspase-mediated apoptosis 34. For example, tetrandrine is an alkaloid from *Radix stephania tetrandrae* that could improve the efficacy of sorafenib by facilitating the production of ROS in human HCC cells, which subsequently promoted caspase-induced cell apoptosis by increasing Bim and decreasing Mcl-1 35. Therefore, AIFCM has been shown to be a promising strategy to enhance the therapeutic efficacy by synergistically inducing apoptosis of cancer cells in combination with chemotherapy.

The mammalian cell cycle is the process involving the progression of the gap 1 (G1) phase, DNA synthesis (S) phase, gap 2 (G2) phase, mitosis (M) phase, and quiescent (G0) phase 36. The development of cancer is characterized by cell cycle dysregulation, which is often caused by the misregulation of cyclin-dependent kinase (CDK) activity 37. In addition to CDKs, the mitotic spindle also plays an important role in cell division and cell cycle, which are two targets of AIFCM in combination chemotherapy. By regulating CDKs and tubulin in the mitotic spindle, AIFCM has been found to cause cancer cell cycle arrest mainly at the G0/G1 phase, S phase or G2/M phase, which could be applied with other chemotherapeutic agents together for inducing cell cycle arrest-mediated cell death and chemotherapy sensitization 38, 39. For example, berberine derived from Chinese medicine *Coptis chinensis* was reported to increase the cytotoxicity of cisplatin on human ovarian cancer cells by inducing G0/G1-phase blockade 40. Moreover, AIFCM can regulate different CDK activities to promote cell cycle arrest in cancer combination therapy. For example, Ho et al. combined cisplatin and triptolide which is a natural product from *Tripterygium wilfordii* to inhibit human bladder cancer, and the combination therapy significantly induced cell cycle arrest in S phase by decreasing the expression of cyclin D1 and cyclin E 41. In a similar study, β-elemene which is a volatile oil isolated from *Rhizoma zeodaria*, was used to enhance the therapeutic efficacy of cisplatin in non-small-cell lung cancer by synergistically inducing cell cycle arrest at the G2/M phase with increased level of checkpoint kinase 2 (CHK2) and reduced activity of cell division cycle protein 2 (CDC2) 42. Besides, AIFCM such as paclitaxel and vincristine can target tubulin and affect spindle function to block the cell cycle 43, 44. Paclitaxel is a clinically approved drug for treating ovarian cancer, breast cancer, Kaposi's sarcoma and non-small-cell lung cancer 45. It can promote the polymerization of tubulin and stabilize the formed microtubules, thus disrupting cell division and inhibiting cell proliferation 44. It was found that the combination of paclitaxel and doxorubicin (DOX) exerted synergistic anticancer effects in H22 tumor-bearing mice. Paclitaxel blocked the cell cycle at the G2/M phase and thus enhanced the synergistic cytotoxicity 46. What's more, clinical trials have been done with paclitaxel and liposomal doxorubicin (PLD) in the weekly treatment of metastatic breast cancer (MBC) patients, showing practical value of this combination therapy in clinical application 47. Vincristine, which is an alkaloid extracted from *Catharanthus roseus*, has been clinically approved for treating a series of cancers, such as acute lymphoblastic leukemia 43. Unlike paclitaxel, vincristine can disrupt tubulin polymerization to inhibit mitosis 48. In one study, vincristine and DOX were co-administered to inhibit tumor growth and achieved significantly better efficacy than single-agent therapy in A549 xenograft mice model. Vincristine in combination chemotherapy was found to efficiently induce cell cycle arrest at M phase when DOX blocked DNA replication, both contributing to cancer cell death 49. Thus, AIFCM can improve the anticancer effect of different chemotherapies by blocking the cell cycle, mainly by acting on CDKs and mitotic spindles.

DNA replication is necessary for the sustained development of tumors 50. AIFCM, such as camptothecin (CPT) and arsenic trioxide, can interfere with DNA replication to promote DNA damage or DNA synthesis impediment caused by chemotherapeutic drugs for efficient combination therapy. For example, CPT is an alkaloid extracted from *Camptotheca acuminata*. The combination of a topoisomerase I (TOP1) inhibitor CPT and a topoisomerase II (TOP2) inhibitor DOX achieved synergistic effects to induce more potent inhibition of tumor proliferation than single-agent treatment 51. The main reason is that CPT can stabilize covalent topoisomerase I-DNA complexes, thus inhibiting DNA synthesis 52. Another example is arsenic trioxide, which comes from natural mineral Arsenolite and has a long history of being used as medicine in China. Arsenic trioxide is now used as a first-line drug for the clinical treatment of acute promyelocytic leukemia, with the application mechanism of causing DNA damage by producing ROS 53. Based on this fact, Liu and colleagues combined DOX and arsenic trioxide for synergistic cancer therapy, in which arsenic trioxide prevented DNA damage repair by inhibiting the activity of PARP-1, thus facilitating the DNA damage caused by DOX 54. Thus, AIFCM holds great potential in synergistic anticancer therapy owing to its promotion effect on the chemotherapy-mediated inhibition of cancer DNA replication.

#### 2.1.2 Reversal of Drug Resistance

Multidrug resistance (MDR) is a major problem for current chemotherapy strategies as it seriously limits the chemotherapeutic effectiveness in clinic, leading to poor prognosis of patients and even treatment failure 55. The factors related to tumor resistance include increased drug efflux and suppressed apoptosis, as well as the tumor microenvironment (TME) 56. Many studies have revealed that AIFCM can regulate these factors to resensitize drug resistant cancer cells to chemotherapy for enhanced therapeutic efficacy 57, 58.

ATP-binding cassette (ABC) transporters are responsible for the increased efflux of hydrophobic chemotherapeutic drugs, such as taxanes and anthracyclines 59. The well-studied ABC proteins, which are overexpressed in cancer cells, include multidrug resistance protein 1 (MDR1, also known as P-glycoprotein (P-gp)), multidrug resistance associated-protein 1 (MRP1) and ABC-family G member 2 (ABCG2, also called BCRP) 60. Targeting these three ABC proteins is a promising approach for decreasing drug efflux and treating drug-resistant cancer with AIFCM combined chemotherapy 61. (1) P-gp was the first identified ABC transporter and is overexpressed in many cancers, including liver, kidney and colon cancers, as well as leukemias and lymphomas 62. Many AIFCM, such as gambogic acid 63, curcumin 64, quercetin 65 and epigallocatechin gallate 66, have been investigated and demonstrated to decrease the expression of P-gp, facilitate the accumulation of chemotherapeutic drugs, reverse the P-gp mediated drug resistance and thus enhance the antitumor effect. For example, many alkaloids have shown P-gp inhibitory activity with their structure of basic nitrogen atom and planar aromatic ring 67. Our works have revealed tetrandrine could efficiently suppress P-gp activity by directing binding in human ovarian cancer cells, and thus enhanced the efficacy of paclitaxel 68. In addition, AIFCM such as osthole, resveratrol and matrine can reduce P-gp expression by inhibiting PI3K/Akt pathway, which could regulate the gene expression of ABC transporters 69-71. For instance, matrine, an alkaloid obtained from the dried root of *sophora flavescens,* was found to reverse the resistance of esophageal carcinoma cells (Eca-109/VCR) to vincristine via suppressing PI3K/Akt/mTOR pathway, resulting in the down-regulation of P-gp, MRP1 and induction of autophagy 69. In addition, some flavone (like apigenin) showed to bind to the C-terminal nucleotide-binding domain (NBD2) of P-gp and compete with its ATP substrate for P-gp inhibition and MDR reversal 72. (2) The overexpression of MRPs such as MRP1, MRP2, and MRP7 is associated with poor chemotherapeutic outcomes in several cancers including breast cancer and lung cancer. Among these MRPs, MRP1 has been widely studied in the development of therapeutic strategies for drug resistant cancer 73, 74. Resveratrol (RSV), which is a polyphenol comes from grape and Chinese medicine *Rhizoma polygoni cuspidati*, was found to downregulate the expression of MRP1 and P-gp in DOX-resistant breast cancer cells, promoting the accumulation of DOX in resistant cells and thus enhancing the effects of chemotherapy in MDA-MB-231/ADR tumor-bearing mice 75. Moreover, resveratrol could also reverse the resistance of human bladder cancer cells pumc-91/ADM to doxorubicin by down-regulating MRP1, lung resistance protein (LRP, mediates drug efflux), Glutathione S-transferase (GST, related to direct detoxification mediated drug resistance), and decreasing the expression levels of Topo-II and Bcl-2 that related to apoptosis resistance 76. In addition to the down-regulation of MRP1 expression, AIFCM such as glaucine, which is an isoquinoline alkaloid from *Corydalis yanhusuo*, could bind to the substrate site of MRP1 and P-gp for competitive inhibition and drug efflux reduction, thus effectively reversing the resistance of MCF-7/ADR cells to adriamycin and mitoxantrone 77. (3) BCRP was the third identified major drug efflux pump in the ABC family, and elevated BCRP levels are associated with treatment failure of chemotherapy in cancers such as acute myelogenous leukemia (AML) and breast cancer 62, 73. Shah et al. reported that curcumin, a polyphenol derived from *Curcuma longa*, downregulated BCRP and increased the cytarabine sensitivity of cancer cells from AML patients 78. Besides, similar to the inhibitory mechanism of P-gp, the inhibition of BCRP can also be mediated by AIFCM through direct inhibition or Regulation of PI3K/AKT pathway 77, 79. For example, nuciferine, an alkaloid from *Nelumbo nucifera* and *Nymphaea caerulea*, down-regulated the P-gp and BCRP levels in the HCT-8/T and A549/T cells that resistant to paclitaxel by inhibiting the upstream nuclear factor erythroid 2-related factor 2 (Nrf2) and hypoxia-inducible factor 1-alpha (HIF-1α) via PI3K/AKT pathway, leading to enhanced tumor suppression effect in A549/T cell xenograft mice with combination treatment 79. Thus, AIFCM are promising agents that facilitate drug accumulation and increase chemosensitivity in MDR tumor cells.

**Figure 2:**
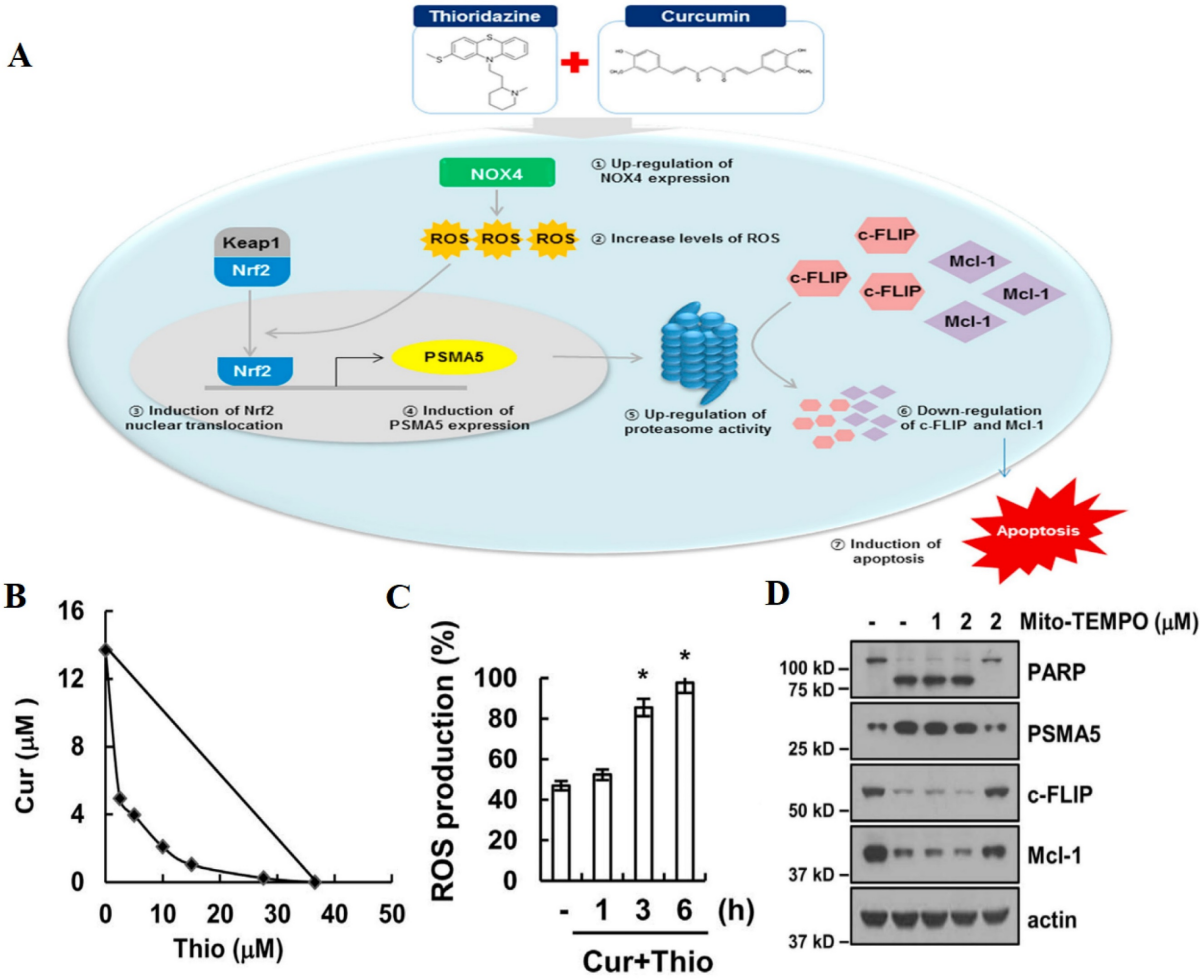
The combination therapy of curcumin and thioridazine for resistant human head and neck squamous cell carcinoma (AMC-HN4). (A) A scheme showing that the combination therapy downregulated the expression of c-FLIP and Mcl-1 via NOX4-mediated ROS production for restoring apoptosis. The synergistically induced (B) apoptosis and (C) ROS production by curcumin and thioridazine in AMC-HN4 cells. (D) The downregulation of c-FLIP and Mcl-1 induced by the combination therapy in NOX4-dependent manner. Reproduced from 87 with permission from Elsevier.

Resistance to apoptosis in cancer cells severely impairs the efficacy of chemotherapy, which is mainly mediated by Bcl-2 protein family, inhibitors of apoptosis proteins (IAPs) and cellular FADD-like IL-1β-converting enzyme-inhibitory protein (c-FLIP) 80. AIFCM has been found to regulate the expression of these apoptosis resistance related proteins and sensitize the cancer cells to chemotherapeutic agents, especially in the treatment of glioblastoma, non-small-cell-lung cancers, and metastatic cancers 81. For example, the high Bcl-2/Bax ratio usually contributes to apoptosis resistance and treatment failure in chemotherapy 82, since the overexpression of anti-apoptotic proteins in Bcl-2 family can promote cancer cell to evade chemotherapy-mediated apoptosis, while the up-regulation of pro-apoptotic proteins would facilitate cancer cell apoptosis for enhanced chemotherapeutic effect. Thus, the decreased Bcl-2/Bax ratio of cancer cells would be beneficial for reversing drug resistance in chemotherapy. Research found that andrographolide, a labdane diterpenoid from *Andrographis paniculata* could prevent Bax degradation, thus facilitating apoptosis and reversing 5-fluorouracil (5-FU) resistance in resistant colorectal cancer cells in which Bax was silenced 83. IAPs are a family of anti-apoptotic proteins that are associated with drug resistance and tumor survival 84. Dhandapani et al. found that curcumin could decrease the expression of IAP family members and Bcl-2 by downregulating their transcription factors nuclear factor κB (NF-κB) and activator protein-1 (AP-1), and thus enhanced cell apoptosis and increase the sensitivity of glioma cells to several chemotherapeutic agents including cisplatin, CPT, and DOX 85. In addition, c-FLIP is a major anti-apoptotic regulator and resistance factor of extrinsic apoptosis 86. In Kwon's work, thioridazine and curcumin were combined for treating human head and neck cancer that was not sensitive to thioridazine alone. The results showed that the combination therapy downregulated the expression of c-FLIP via NADPH oxidase 4 (NOX-4)-mediated ROS production (Figure 2) 87. Therefore, AIFCM can reverse apoptosis inhibition to facilitate the efficacy of chemotherapeutic drugs in MDR cancer cells.

The TME can protect cancer cells from the cytotoxic effects of chemotherapeutic agents, leading to the development of drug resistance 88. The extracellular matrix and stromal cells especially cancer-associated fibroblasts in TME contribute to the induction of drug resistance of cancer cells by promoting angiogenesis, hypoxia and regulating cytokine and growth factor expression. Research has found that AIFCM could regulate the factors associated with the TME-related drug resistance, including hypoxic conditions, integrins, cytokines, and growth factors, which helps to improve the effect of chemotherapy 89. Owing to the fast and uncontrolled proliferation of neoplastic cells, the tumor parts far away from blood vessels commonly develop hypoxia, which has been found to negatively affect chemotherapeutic outcomes, especially in advanced metastatic cancer 90. Wang et al. reported that 20 (R)-ginsenoside extracted from ginseng could inhibit hypoxia-induced epithelial-mesenchymal transition (EMT) and stemness, improving the efficacy of cisplatin in resistant lung cancer cells 91. In addition to the hypoxic state, integrins, which are cell surface molecules in tumor and stromal cells that can induce cell adhesion to the extracellular matrix, have a profound effect in promoting drug resistance and tumor survival 92. In recent work, curcumin was applied to downregulate the expression of integrin α_v_β_3_ in resistant colon cancer cells and enhance the efficacy of erlotinib 93. In addition to cell surface molecules, dysregulated cytokine and growth factor expression also promote the development of drug resistance 94, 95. Recently, quercetin, a flavonoid from *Bupleurum* was found to modulate the TME and improve the efficacy of temozolomide by significantly decreasing the levels of interleukin-8 (IL‐8), IL‐6, and vascular endothelial growth factor (VEGF), which are related to the drug resistance of glioblastoma cells 96. Thus, it is supposed that AIFCM could reverse drug resistance by regulating TME-related factors.

Over the past few decades, a great deal of research has been carried out to develop drugs to overcome MDR in chemotherapy. However, some drugs, such as p-gp inhibitors and MRPs inhibitors, are still in preclinical research or clinical trials, and have not been approved for cancer treatment due to poor efficacy and high toxicity 97, 98. Therefore, efficient MDR reversal agents with low toxicity still need to be developed. Among those approaches for reversing MDR, utilizing FDA-approved drugs with the ability to overcome MDR for combination therapy with chemotherapeutic drugs has become a promising strategy, especially for chemotherapy-refractory and relapsed cancers. AIFCM usually has the characteristics of low toxicity and small adverse reactions. For example, tetrandrine and curcumin have shown good tolerance and feasibility in clinical trials for patients with drug resistant acute myelogenous leukemia and pancreatic cancer, respectively 99, 100. Therefore, the application of AIFCM combined with chemotherapy shows great research significance and explores the potential for overcoming MDR and improving the therapeutic efficacy.

#### 2.1.3 Suppression of Tumor Metastasis

Tumor metastasis is responsible for the greatest majority of cancer-related deaths. Metastasis is a complicated process that involves the detachment of metastatic cells from the primary tumor, transendothelial migration into lymphatic vessels or blood vessels, adhesion to the target organ site, and development of secondary tumors at the target site 101. In many cases, single-agent chemotherapy cannot inhibit cancer metastasis owing to the complexity of the process 102. Some traditional Chinese herbal compounds such as Huangci Granule (consists of *Ligustrum lucidum*, *Cistanche deserticola*, *snakeberry*, *edible tulip*, *Salvia miltiorrhiza Bge*, and *Fruit of Fiverleaf Akebia*), LiuJunZi decoction (consists of *Codonopsis*, *Atractylodes macrocephala*, *Poria cocos*, *licorice*, *tangerine peel* and *pinellia ternata*) have been proved to relieve symptoms, enhance curative effect, prevent tumor metastasis and recurrence in the treatment of patients with lung cancer, colon cancer, breast cancer or other cancer when combined with conventional chemotherapy 103-105. Various AIFCM show the ability to prevent tumor growth and metastasis 106-108. AIFCM combined with chemotherapy would facilitate the antitumor chemotherapeutic effects. In the development of invasive and metastatic tumors, EMT plays a critical role and represents a biological process in which polarized epithelial cells undergo multiple changes to transform into migratory mesenchymal-like cells with enhanced invasiveness 109. AIFCM could participate in the regulation of the EMT process by an array of signaling pathways associated with the initiation and maintenance of EMT, such as the transforming growth factor-β (TGF-β), PI3K/AKT and Wnt signaling pathways related to tumor metastasis. Among these signaling pathways, TGF-β signaling pathway plays a dominant role in the EMT process. The binding of TGF-β ligand with TGF-β receptor activates downstream PI3K/AKT pathway and Wnt pathway, and then increases the expression of EMT activators, such as phosphorylated Smad2 and β-catenin, to induce and maintain EMT 110. In one study, curcumin was reported to reverse DOX-induced EMT in the treatment of triple-negative breast cancer by decreasing the phosphorylation of Smad2 and the level of β-catenin via the TGF-β and PI3K/AKT signaling pathways, leading to the enhanced anti-proliferation effect of DOX in cancer cells 111. In addition, the blockade of the Wnt signaling pathway by AIFCM could contribute to the downregulation of β-catenin and the inhibition of EMT 110. For example, the combination of tetrandrine and 5-FU was found to exhibit synergistic antitumor effects and efficiently inhibit the migration and invasion of human colorectal cancer cells by inactivating the Wnt/β-catenin pathway 112.

Apart from inhibiting the EMT process, regulating tumor-related biological conditions is also a strategy for AIFCM to inhibit tumor metastasis 113, 114. For example, angiogenesis plays essential role in the metastatic growth of cancer cells, the hypoxia and VEGF-induced changes in angiogenesis would promote the invasion and metastasis of tumor cells 115. Thus, the AIFCM-mediated angiogenesis inhibition combined with chemotherapy could aid in the inhibition of tumor metastasis. Some AIFCM such as andrographolide, curcumin and triptolide have shown anti-angiogenic activity by inhibiting VEGF-induced HUVEC migration and invasion 116-118. Wong's group found that andrographolide decreased the formation of human umbilical vein endothelial cell (HUVEC) tubes and downregulated the expression of VEGF, VEGF-R2 and CD31 in tumor tissue. The combination of andrographolide and DOX exerted potent inhibitory effect on breast cancer and anti-metastasis effect in lung tumor 116. Similarly, our group reported that curcumin combined with DOX could inhibit the proliferation, migration and invasion of HUVEC more effectively than single-drug treatment 117. Moreover, cancer stem cells (CSCs) are believed to initiate the development of tumor metastasis 119. Yang et al. combined paclitaxel and curcumin for cancer therapy, which showed significant inhibition on the proliferation of both non-CSCs and CSCs, and thus efficiently prevented tumor metastasis 120. Therefore, AIFCM have the potential to prevent tumor metastasis to facilitate chemotherapy by inhibiting EMT via different signaling pathways and regulating tumor biological conditions. Among these AIFCM, a variety of polyphenols such as resveratrol, genistein have been widely researched and shown excellent anti-angiogenic and anti-metastatic ability. These polyphenols are usually natural antioxidants present in vegetables, fruits and foods such as grapes and soybeans. They can reduce the risk of cancer by participating in people's daily diet, and can inhibit tumor angiogenesis by inhibiting VEGF, matrix metalloproteinase-2 (MMP-2) and HIF-1α for further exhibiting anti-proliferative and anti-metastatic effects 121. Especially curcumin which could increase the expression of anti-metastatic proteins, improve the effectiveness of chemotherapy and enhance the survival rate of cancer patients in clinical trials, exerts a broad-spectrum anticancer activity and good clinical application prospect 122.

In conclusion, AIFCM have shown the advantages of low toxicity and few side effects, and exhibited a variety of therapeutic functions and pharmacological effects to promote the efficacy of chemotherapy. First, AIFCM can inhibit tumor proliferation and enhance the efficacy of chemotherapy by inducing cell apoptosis, blocking cell cycle and interfering DNA replication. Second, AIFCM can also reverse multidrug resistance by regulating the expression of ABC transporters and multiple signaling pathways such as Bcl-2, NF-κB, and PI3K, thus sensitizing the tumor to chemotherapeutic drugs, reducing the side effects, and improving the efficacy of chemotherapy. Third, AIFCM can prevent tumor metastasis and recurrence by inhibiting EMT, angiogenesis and CSCs proliferation. Thus, AIFCM has been proved to be effective natural adjuvants and sensitizers for enhancing chemotherapy. However, considering that AIFCM usually exerts the function of chemotherapy improvement by maintaining synergistic anti-tumor effects with chemotherapy agents, the potential drug interactions between those components in the combination therapy should be taken into account 123. For example, curcumin and artesunate have been found to cause side effects or toxicity in combination with chemotherapeutic agents 124, 125. What's more, some AIFCM such as curcumin, resveratrol and honokiol tend to have the abilities to induce multiple models of cell death, including necrosis, ferroptosis, or autophagy, which also helps to overcome chemotherapy resistance 126. Thus, the targets and molecular mechanisms need to be further clarified when applying AIFCM in combination chemotherapy. Additionally, since some AIFCM with multi-target properties lack the selectivity against tumor cells, it is necessary to improve their selectivity through cancer-targeted nanocarriers or chemical modifications.

### 2.2 AIFCM Combined with Gene Therapy

**Figure 3:**
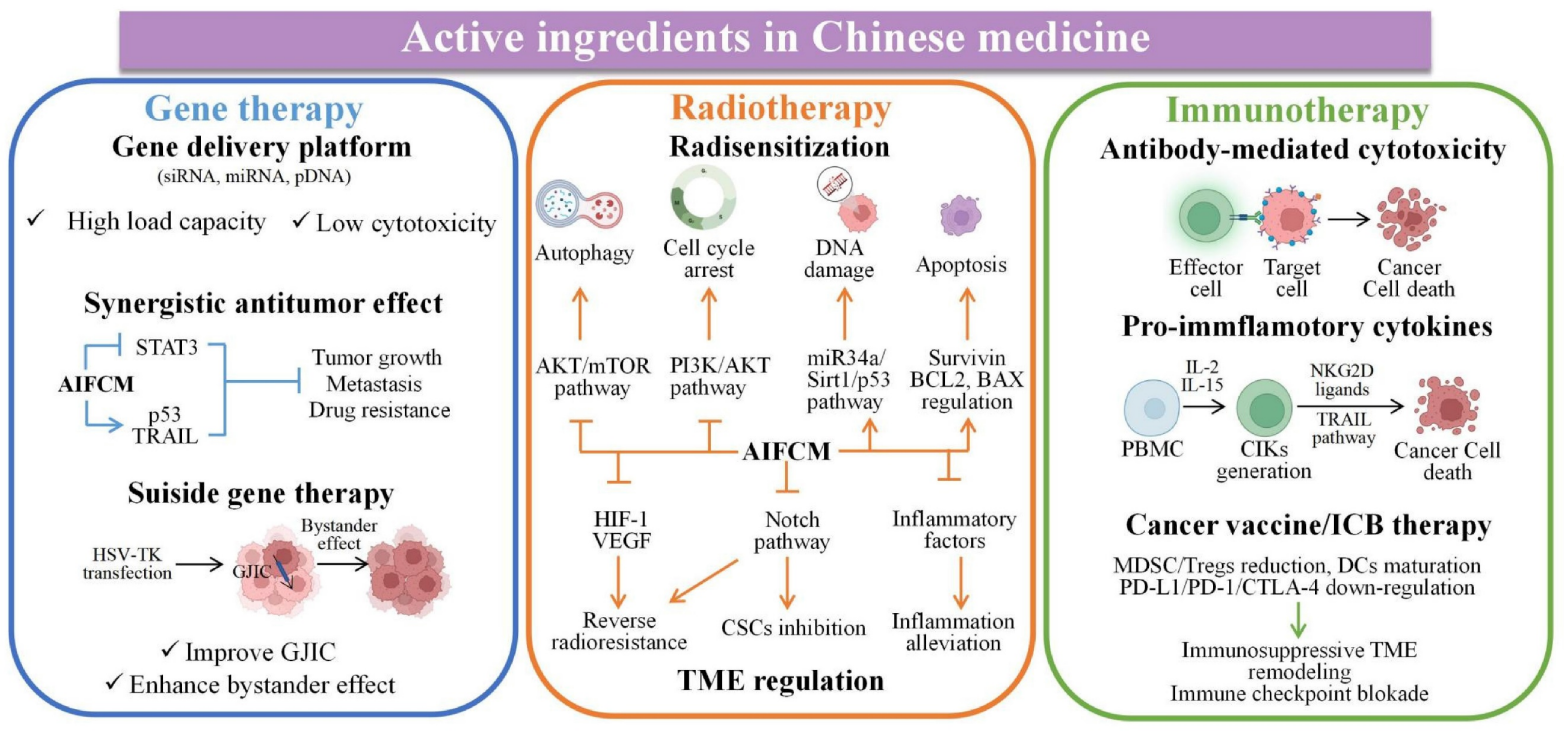
The function and mechanism of AIFCM enhancing the therapeutic effect of gene therapy, radiotherapy and immunotherapy in combination therapy.

**Figure 4:**
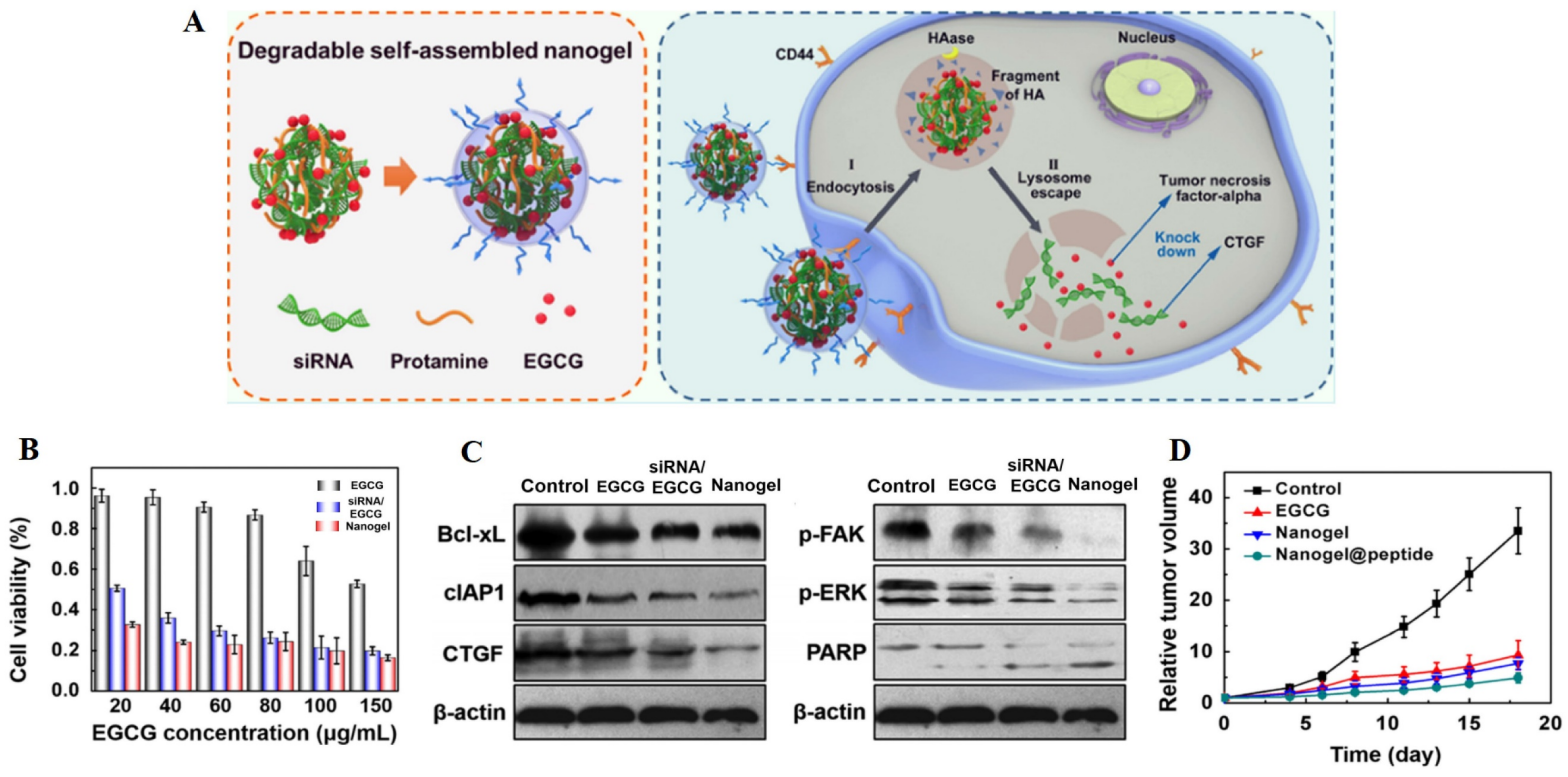
Combination of EGCG and CTGF siRNA for inhibition of resistant breast cancer. (A) Schematic illustration of the combination therapy for inhibiting CTGF-overexpressed breast cancer. (B) The enhanced cytotoxicity by the combined treatment in MDA-MB-231 cells. (C) Downregulation of drug resistance-associated proteins in MDA-MB-231 cells by the combination therapy. (D) The reduced tumor volume on xenografted mice by the combined treatment. Reproduced from 131 with permission from American Chemical Society.

In chemotherapy, tumor heterogeneity as well as the complexity of the related signaling pathways or targets of tumor treatment have brought challenges to the effectiveness of chemotherapeutic drugs. In order to address these barriers to achieve satisfactory therapeutic efficacy in cancer treatment, various novel therapies have been developed. Among them, gene therapy can be used to deliver nucleic acids to inhibit the expression of mutated genes or to facilitate the expression of deleted or down-regulated proteins, which can make up for the deficiencies of chemotherapeutic drugs against multiple anti-tumor targets, and is considered as a promising strategy for eliminating tumors 127. Different kinds of nucleic acids, mainly including small interfering RNA (siRNA), microRNA (miRNA) and plasmid DNA (pDNA), have been combined with AIFCM to eradicate cancer. In these combination therapies, nucleic acids can promote cell apoptosis, modulate drug resistance or inhibit tumor metastasis, which would enhance the antitumor effects of AIFCM 128, 129. Moreover, some AIFCM have been widely applied for gene delivery platforms that have better load capacity and lower cytotoxicity in comparison with traditional polymeric nanocarriers due to their high binding affinities with various kinds of nucleic acids (Figure 3). In particular, polyphenols show an advantage for gene delivery, as they can bind to various biomolecules including nucleic acids with a high affinity by non-covalent interactions 130, 131. For examples, Zhu's group encapsulated a kind of siRNA with protamine and EGCG, the major catechin in tea, which could attach to the siRNA chains and help to form degradable carriers. After the siRNA and EGCG release, the chemosensitivity of resistant breast cancer cells could be significantly increased by silencing the overexpressed connective tissue growth factor (Figure 4) 131. Besides EGCG, a variety of natural polyphenols such as polycatechols have also been developed for siRNA delivery polymers with high delivery efficiency 130. Additionally, AIFCM could combine with gene therapy to inhibit tumor growth, overcome drug resistance and combat metastasis for enhanced synergistic anticancer effect through similar signaling pathways and antitumor targets 132-134. As a typical example, regarding the tumor necrosis factor-related apoptosis-inducing ligand (TRAIL) plasmid (pTRAIL) has been proven to induce tumor apoptosis, Wang et al. combined pTRAIL and gambogic acid to inhibit intractable triple-negative breast cancer tumor growth. The combination therapy was found to induce more cell apoptosis (93.1%) than gambogic acid (55.92%) or pTRAIL (17.35%) alone 132. Furthermore, Jose et al. synthesized the curcumin and signal transducer and activator of transcription 3 (STAT3) siRNA co-loaded cationic liposomes for treating skin cancer. The STAT3 inhibition mediated by curcumin combined with the STAT3 silencing could synergistically suppress tumor growth, which showed better therapeutic efficacy than using single therapy 134. Similarly, Xu et al. combined resveratrol, which could elevate the transactivation of p53 activity, with p53 gene via liposomes for treating Hela and MCF-7 cells. This synergistic treatment could help to reduce the toxic side effect of resveratrol and improve the anti-cancer efficiency of p53, leading to improved therapeutic results 133.

Besides, AIFCM could enhance the efficacy of the suicide gene therapy mediated by herpes simplex virus-thymidine kinase (HSV-TK), in which the TK gene transfection would induce the generation of toxic metabolites from the substrate ganciclovir (GCV) in cells, leading to cell death and the bystander killing effect of surrounding TK-negative cells. The efficacy of suicide gene therapy is unsatisfactory in some types of cancer, such as hepatocellular carcinoma and melanoma, which have poor gap junction intercellular communication (GJIC) function and the resulting reduced bystander killing effect. AIFCM such as resveratrol and curcumin have been found to up-regulate the gap junction proteins such as connexin 43 and connexin 32 in cancer cells, improve GJIC, and thus enhance the killing effect and bystander effect of suicide gene therapy 135, 136.

Increasing evidence suggest that gene therapy could help to overcome drug resistance of cancer cells to some AIFCM and improve their anticancer activity. For example, the Nur77/ΔDBD gene was reported to downregulate stanniocalcin 2 (STC2) overexpression in hepatocellular carcinoma, which increased the expression of P-gp and Bcl-2. When combined with paclitaxel, the Nur77/ΔDBD gene efficiently inhibited the paclitaxel efflux induced by P-gp and decreased anti-apoptotic Bcl-2 expression and increased pro-apoptotic protein expression, thus improving the antitumor effects of paclitaxel on paclitaxel-resistant hepatoma 137. In addition, miRNAs such as miR-33a-5p or miR-34a could also be used to reverse drug resistance of AIFCM in multiple kinds of cancer. Li et al. reported that miR-33a-5p, an inactivated tumor suppressor gene in lung cancer, increased the sensitivity of lung adenocarcinoma to celastrol by acting on its target mRNA of mammalian target of rapamycin (mTOR) 138. In the study of Kang et al., they found that miR-34a significantly inhibited the stemness and mammosphere formation of breast cancer cells by downregulating neurogenic locus notch homolog 1 (Notch1). The combination of miR-34a and paclitaxel efficiently inhibited the progression and metastasis of breast cancer 139.

In summary, the combination therapy of AIFCM and gene exhibits improved synergistic anticancer effects. By regulating the gene expressions (such as TRAIL, STAT3 and p53) and the related signaling pathways, the proposed combination therapy can exert synergistic effects in inhibiting treatment resistance, tumor growth and metastasis. It's worth noting that, the delivery of nucleic acids including siRNA, miRNA and pDNA to tumor cells usually needs the help of nanocarriers, which could reduce off-target effects and adverse effects, and enhance therapeutic outcomes 14. Therefore, the co-delivery of AIFCM and nucleic acid needs further research to achieve the ideal synergistic antitumor effect with the combination of nanotechnology.

### 2.3 AIFCM Combined with Phototherapy

**Figure 5:**
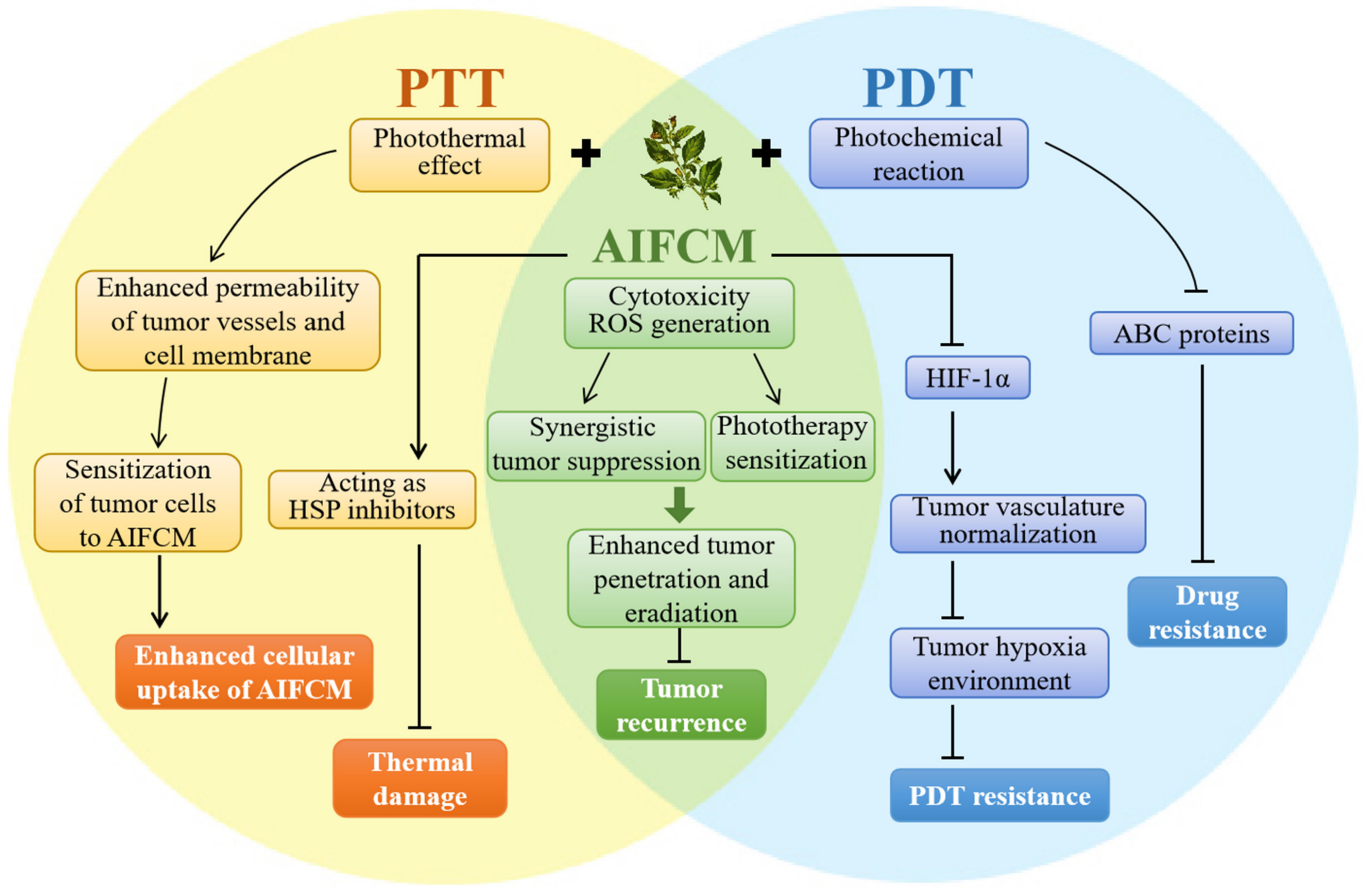
The anticancer mechanism of AIFCM combined with PTT, PDT, or PTT and PDT modalities for tumor therapy.

Phototherapeutic modalities are activated by light irradiation and produce heat (photothermal therapy, PTT) or ROS (photodynamic therapy, PDT) to induce tumor cell death 140. As light irradiation and dosing regimens are easily adjustable based on the patients' tolerance, phototherapy is supposed to be a minimally invasive, direct and accurate therapeutic approach for cancer 141. However, because the light penetration depth in phototherapy is limited and the therapeutic modality would only cause local damage at the target tumor site, phototherapy alone usually exhibits insufficient tumor penetration depth and leads to incomplete tumor eradication 142. Currently, the near-infrared light (NIR-I, 750-900nm; NIR-II, 1000-1700 nm) absorbing materials are used in phototherapy to improve the tissue penetrating depth, but some problems such as tumor recurrence, therapeutic resistance and side effect still exist in cancer treatment with phototherapy 143. AIFCM have been proven to solve these problems and achieve better anticancer outcomes when combined with phototherapy. In combination therapy, AIFCM can not only exert cytotoxic effects, and reduce tumor recurrence, but also overcome the limited therapeutic effect of phototherapy by increasing the sensitivity of cancer cells. In particular, AIFCM such as quercetin, and gambogic acid can reduce the thermal damage of normal tissues and achieve mild photothermal effects in PTT. Also, some AIFCM can improve tumor hypoxia and enhance therapeutic effects in PDT (Figure 5). The following sections discuss the recent development of AIFCM combinations with PTT, PDT, or PTT and PDT modalities for tumor therapy.

#### 2.3.1 AIFCM Combined with PTT

PTT relies on the photothermal effect of photothermal agents to convert the light energy into heat to increase the temperature of the tumors well above the physiological temperature for several minutes to cause irreversible damage to tumor tissue 144. Different kinds of PTT modalities (both organic fluorescent molecules and inorganic nanoparticles) with good photothermal conversion ability have been combined with AIFCM for combating cancer.

**Figure 6:**
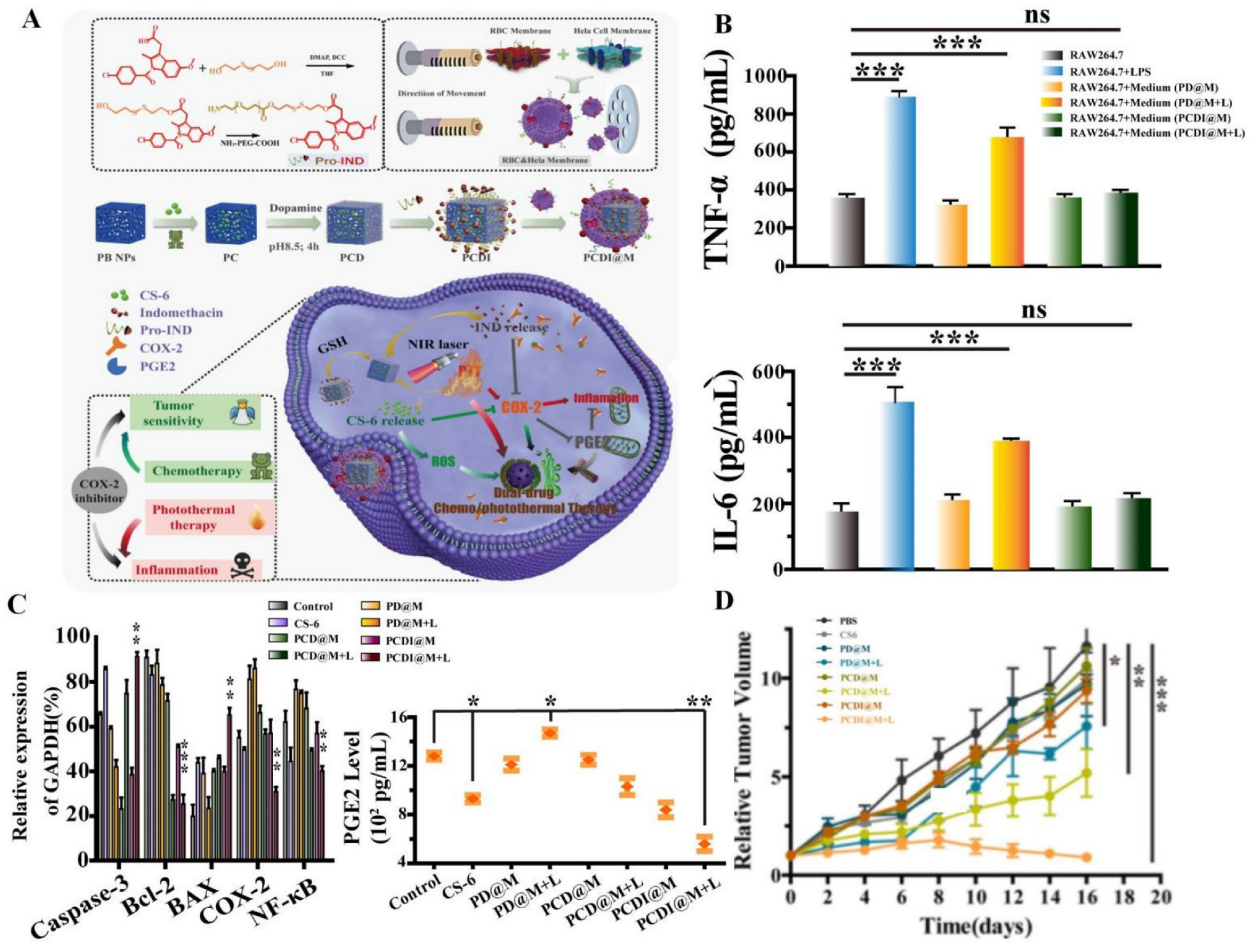
Combination of photothermal therapy and chemotherapy based on gamabufotalin and indomethacin. (A) A scheme showing the suppression of PTT-induced inflammatory response and chemotherapeutic sensitization effect by gamabufotalin and indomethacin via COX-2/PGE2 pathway. (B) The combination therapy down-regulated the PTT-induced pro-inflammatory cytokines (TNF-α and IL-6) of Hela cells. (C) The combined treatment inhibited the COX-2/PGE2 pathway and IKK/NF-κB pathway, and decreased the Bcl-2/Bax ratio in Hela cells. (D) The *in vivo* synergistic tumor inhibition by the combined treatment. Reproduced from 148 with permission from Elsevier.

Research found that AIFCM could combine with various kinds of fluorescent materials, such as polypyrrole and IR780 iodide that would produce heat under NIR light irradiation in PTT, for overcoming the obstacle of incomplete removal of cancer cells, leading to better tumor elimination effect and lower tumor recurrence rate 145, 146. For example, the combination of polypyrrole and paclitaxel achieved enhanced tumor inhibition efficiency and synergistic antitumor effect, which contributed from the photothermal effect of polypyrrole under 808 nm irradiation and potent cytotoxicity of paclitaxel in lung cancer 145. In addition, Shi et al. used IR780 and CPT to achieve combination anticancer therapy for cervical cancer. Under the hyperthermia generated by IR780, the cellular uptake of CPT significantly increased and thus the tumor recurrence rate declined, which could synergistically lead to complete tumor eradication with no observed recurrence 147. However, the thermal diffusion or overheating in PTT process could cause undesirable necrosis and thermal damage of the surrounding normal tissues, resulting in inflammation associated with tumor growth and metastasis. To solve this problem, AIFCM like gamabufotalin with anti-inflammatory and anti-cancer activity can be applied in PTT for suppressing inflammatory response and promoting therapeutic effect. Xiao et al. used gamabufotalin and anti-inflammatory drug indomethacin in combination with PTT for cervical cancer treatment. These two drugs synergistically inhibited COX-2/PGE2 pathway, down-regulated the level of inflammatory cytokines (TNF-α and IL-6) in Hela cancer cells, and suppressed the PTT-induced inflammatory response, leading to enhanced therapeutic effect and biosafety of combined chemo-and photothermal therapy (Figure 6) 148. In contrast, insufficient heating often exhibits limited tumor inhibition due to the thermoresistance induced by heat shock proteins (HSPs) 149. AIFCM such as quercetin and gambolic acid could act as HSP inhibitors for mild-temperature PTT, which may be a promising therapeutic approach in cancer treatment. For example, Ali et al. used quercetin as HSP70 inhibitor to enhance the photothermal therapeutic effect of gold nanorods under NIR irradiation, leading to cancer cell death mainly by apoptosis instead of necrosis at lower temperature (<50 °C) 150. In our recent work, we combined indocyanine green (ICG) and gambogic acid, which is a xanthonoid from *Garcinia hanburyi* and can act as HSP90 inhibitor, to induce low-temperature PTT to eradicate breast cancer. Under mild NIR light irradiation, this combination exhibited efficient tumor destruction and did not cause damage to normal tissue when the temperature rose to 43 °C 149.

Additionally, the photothermal effect of PTT would elevate the permeability of tumor vessels and cancer cell membrane, leading to enhanced sensitivity of tumor cells to AIFCM. Specifically, the high temperature mediated by PTT could increase the blood flow and microvascular pore size of tumor, and ensure high kinetic energy of membrane phospholipids as well as high fluidity of cell membrane 151, 152. These features could be accountable for the enhanced drug delivery to tumor cells in PTT. Thus, PTT has been used to realize enhanced release efficacy and cellular uptake of AIFCM in combination therapy, which could realize satisfying therapeutic effects. Metal organic nanoparticles (NPs), transition metal oxides and dichalcogenides with high absorption in the NIR region and excellent photothermal conversion efficiency, such as molybdenum oxide nanoparticles, Ag and Au nanoparticles, have been widely exploited for combination therapy with AIFCM 153, 154. For instance, Bao and coworkers synthesized polyethylene glycol (PEG)ylated MoO_3-x_ hollow nanospheres with high NIR absorption, which makes them promising PTT agents and photoacoustic tomography (PAT) imaging agents. The combination of MoO_3-x_ NPs and CPT achieved incredible therapeutic outcomes against pancreatic tumor-bearing mice (nearly 95% inhibition) when CPT alone led to cell death in only half of the treated cells 153. In addition to metal NPs, Pang et al. combined graphene oxide (GO), an excellent carbon material, with the artemisinin derivative artesunate to combat liver cancer. GO exhibited an efficient PTT effect that facilitated the cellular uptake of artesunate and the generation of peroxynitrite, which is a critical substance related to artesunate toxicity. As a result, the combination completely eradicated tumors *in vivo* due to the chemo-photothermal synergistic effect 155.

Accordingly, numerous studies that combined PTT modalities and AIFCM for tumor therapy have achieved improved therapeutic outcomes. In summary, the synergistic effect could be attributed to the heat generated from PTT and the cytotoxic effect of AIFCM. On the one hand, some AIFCMs can reduce the tumor recurrence rate in PTT, increase the sensitivity of PTT to cancer cells, and reduce the adverse side effects caused by high-temperature photothermal effect, so that providing opportunities for mild-temperature PTT to be used for tumor therapy. On the other hand, the PTT can kill tumors by thermal ablative approaches, and increase the cellular uptake of AIFCM in tumor tissue, which improves the efficacy of AIFCM and may help to overcome the MDR of them. In the future, more studies should be focused on identifying AIFCM with the ability to reverse thermal resistance in cancer cells to facilitate PTT.

#### 2.3.2 AIFCM Combined with PDT

In PDT protocols, tissue-penetrating light is utilized to irradiate cancer cells/tumor tissue, which selectively take in certain photosensitizers, and then the photosensitizer is activated to produce ROS, mainly singlet oxygen, to cause tumor inhibition. Generally, photosensitizers that have been widely used in tumor therapy can be categorized into two types, porphyrins and nonporphyrins 156. However, similar to PTT, the penetration depth of light that triggers photosensitizers is limited, and consequently, only the local tumor cells could be eliminated. Moreover, the ROS generation capacity of PDT is insufficient and is limited by the hypoxic tumor environment. Therefore, different approaches have been developed to conquer these obstacles for more effective PDT and promote its clinical applications. Research shows that AIFCM can be used in combination with photosensitizers to expand the therapeutic range and treatment effect by exerting its ROS generation ability and anti-tumor cytotoxicity for synergistic cancer therapy. Moreover, some AIFCMs such as CPT, and dihydroartemisinin have the ability to relieve tumor hypoxia or overcome PDT resistance, which also improve the effect of PDT combination therapy 157.

Porphyrins are a group of tetrapyrrolic molecules that play a dominant role in inducing PDT 158. Many kinds of AIFCM like triptolide, astragaloside III, and camptothecin have been exploited in combination with porphyrins and their derivatives mediated PDT to synergistically combat cancer, relieve the tumor hypoxia environment and overcome the resistance of tumor cells to drug or PDT 159, 160. For example, Chen et al. combined the synergistic effect of porphyrin and CPT to treat metastatic colorectal cancer, resulting in highly inhibiting tumor growth and recurrence. In this combination therapy, the photodynamic effect induced by porphyrin effectively inhibited the expression of adenosine-triphosphate (ATP)-binding cassette subfamily G member 2 (ABCG2) that associated with drug resistance, and thus facilitated the therapeutic effects of CPT. Moreover, CPT reduced the hypoxia-responsive HIF-1α expression and normalized the tumor vasculature, which help to overcome the hypoxia-induced drug resistance in PDT combination therapy 161. Similarly, Zhang et al. combined another porphyrin derivative, 5,10,15,20-tetro (4-pyridyl) porphyrin (H_2_TPyP), and curcumin to inhibit non-small cell lung cancer. According to the FRET effect with perylene, H_2_TPyP could emit NIR fluorescence for imaging and produce singlet oxygen to kill cancer cells, leading to enhanced tumor inhibition with the chemotherapeutic effect of curcumin 162. Moreover, AIFCM could exert their antitumor cytotoxicity and promote tumor elimination efficacy in PDT-resistant tumor. For example, Liu's group combined CPT and a well-investigated porphyrin derivative, chlorin e6 (Ce6), to eradicate human cervical carcinoma when Ce6 was activated to produce ROS to kill cancers and CPT caused DNA topoisomerase-I inhibition and cell death in PDT-resistant cancer cells 163.

Unlike porphyrins, which are heterogeneous in nature, nonporphyrin fluorescent dyes are defined molecules with fewer side effects 156. The combination of nonporphyrin dyes and AIFCM with potent cytotoxicity and ROS generation ability has also achieved enhanced antitumor efficacy. In a work by Liu's group, the photosensitizer eosin Y and CPT were combined to eradicate liver cancer 164. The combination therapy achieved excellent outcomes: eosin Y produced sufficient singlet oxygen by energy transfer with upconversion nanoparticles under 980 nm laser irradiation, and CPT exerted potent cytotoxic effects. In addition to organic NIR dyes, AIFCM could also cooperate with some inorganic molecules for promoted PDT efficiency. For examples, AIFCM has been applied with fullerene (C60) or Ir (III) compounds that would produce ROS synergistically under light irradiation, to increase ROS levels in PDT for cancer treatment. In the study of Zhang and coworkers, they combined C60 and artemisinin, a sesquiterpene lactone derived from *Artemisia annua*, to inhibit breast cancer. The photodynamic effect of C60 upon 532 nm laser irradiation and the cytotoxicity of artemisinin both induced ROS generation and exerted a potent tumor inhibition effect 165. Notably, oxygen plays a critical role in the production of ROS induced by most PDT agents. However, oxygen consumption in the process would weaken the efficacy of PDT, especially in the tumor hypoxia environment 166. Surprisingly, some agents could generate ROS without oxygen. For example, Xiang et al. combined phosphorescent Ir(III) compounds with CPT to combat human cervical cancer. ROS were generated by Ir(III) compounds upon visible light irradiation in an oxygen-independent manner, and CPT exhibited powerful killing effects on cancer cells 167.

The combination of PDT and AIFCM has achieved improved anticancer outcomes. However, in most cases, the generation of ROS by irradiating PDT agents requires oxygen. Oxygen consumption leads to hypoxia in tumor tissue, which further limits the efficacy of PDT and leads to incomplete tumor ablation. The existence of hypoxic regions in solid tumors also severely affects the therapeutic effects of PDT 168. Therefore, it is promising to apply AIFCM which has ROS generation ability and hypoxia-relieving effect to overcome hypoxia-induced resistance and facilitate PDT in combination therapy.

#### 2.3.3 AIFCM Combined with PTT-PDT

Currently, single PTT and PDT therapy are mostly suitable for superficial tumors, such as skin cancer, oral cancer, and esophageal cancer, and cannot completely eliminate the tumor, which poses a risk of tumor metastasis and recurrence. Therefore, AIFCM have been combined with both PTT agents and PDT agents for multifunctional anticancer therapy with enhanced therapeutic effect. The multifunctional therapy exerts its effects via the cytotoxicity of AIFCM, the heating effect of the PTT agent and the ROS-induced damage of the PDT agent, which therefore results in more potent tumor inhibition than single-agent treatment and shows advantage for inhibiting tumor metastasis and preventing tumor recurrence. For example, Zeng et al. utilized gold nanorods as PTT agents, porphyrinic metal-organic frameworks as PDT agents and CPT as a chemotherapeutic agent to treat breast cancer. The combination therapy achieved more significant inhibition efficacy of tumor growth and metastasis than single-agent therapy 169. In addition, some PTT agents with both ROS and heat generation ability under light irradiation have also been used in AIFCM combined therapy to address the therapeutic complexity associated with the use of different excitation lights for PTT and PDT drugs.

For instance, Hou et al. combined mesoporous copper sulfide (CuS) NPs (CuS NPs) and artesunate for synergistic therapy against breast cancer. Upon NIR laser irradiation, CuS NPs increased the temperature and generated ROS to kill cancer cells when artesunate exhibited potent cytotoxicity, leading to a higher tumor inhibition rate in comparison with single therapy 170. In another study, NIR dye ICG and paclitaxel were combined for imaging-guided therapy against non-small-cell lung cancer. ICG could produce ROS and increase the temperature upon laser irradiation to inhibit tumor growth, while paclitaxel exerted its antitumor cytotoxicity. The synergistic combination of ICG and paclitaxel successfully eradicated xenograft tumors with no recurrence and no significant systemic toxicity 171. Thus, PTT-PDT agents and AIFCM can be combined to inhibit cancer and achieve excellent outcomes.

In conclusion, AIFCM with PTT/PDT could induce ROS generation and cytotoxicity in cancer cells, thus realizing a synergistic antitumor effect. This can expand the effective therapeutic depth, and reduce the possibility of tumor metastasis or recurrence. For example, some AIFCM (e.g., triptolide, CPT and dihydroartemisinin) with PDT can inhibit tumor hypoxia and treatment resistance by down-regulating HIF-1α and promoting ROS generation 157, 159, 160. They can be possible candidates for natural photosensitizers in PDT, and show low or no toxicity to normal cells 172. They can also reduce the side effects induced by PTT/PDT, such as the PTT-mediated thermal damage to normal tissue and inflammatory responses. On the other hand, some problems limit the efficacy of PTT/PDT. For example, HSPs and autophagy can mediate two defensive mechanisms for protecting cellular proteins and cancer cells from heat generated by photothermal effect, causing thermoresistance in PTT. Even though AIFCM such as quercetin and gambolic acid can act as HSP inhibitors to relieve thermoresistance 173, the studies of AIFCM as autophagy inhibitors are still very limited. More importantly, clinical studies for the combination therapy of AIFCM and PTT/PDT are necessary to better validate the corresponding therapeutic effect and reactions.

### 2.4 AIFCM Combined with Radiotherapy

Radiotherapy utilizes ionizing irradiation to treat cancer based on the fact that fast-proliferating cancer cells are more sensitive to DNA damage induced by irradiation than normal cells 174. By using deeply penetrating radiation, radiotherapy is able to destroy deep-seated tumors inside the body or internal organs. With this characteristic, radiotherapy is applied as a frontline therapy and is used to treat approximately 50% of newly diagnosed cancers as well as recurrent cancer 175. However, radiotherapy can cause incomplete tumor ablation and trigger a response in the TME, which may promote radioresistance and tumor metastasis 176. A series of AIFCM have been found to enhance the efficacy of radiotherapy by inducing cancer cell death via alternative pathways and regulating the TME to reverse radioresistance, improve radiosensitization and inhibit tumor metastasis 177, 178. Moreover, various kinds of Chinese medicine formulations like Gan Lu Yin (consisting of *Liriope spicata, Citrus sinensis, Scutellaria baicalensis, Rehmannia glutinose, Dendrobium nobile, Glycyrrhilza uralensis, Eriobotrya japonica, Artemisia capillaris* and* Asparagus cochinchinensis)* have shown to reduce adverse side effects such as xerostomia and myelosuppression in cancer patients under radiotherapy 179-181. Also, Chinese medicine like *Selaginella* elevated the remission effect of primary lesions in nasopharyngeal carcinoma patients (30 g daily in the entire or late course of radiotherapy), showing a potential radiosensitization effect 182. Thus, AIFCM-adjutant radiotherapy seems to be a promising approach in cancer treatment (Figure 3).

Research found that AIFCM could combine with radiotherapy for realizing radiosensitization and enhanced tumor inhibition efficiency, which can be mediated by autophagy or apoptosis activation 183. In a work by Yang's group, arsenic trioxide improved the efficacy of radiotherapy in glioma cells by inducing cell cycle arrest and autophagy via inhibiting the PI3K/AKT pathway and activating the ERK1/2 pathway 184. Similarly, Yang et al. found that gambogic acid could also enhance the efficacy of radiotherapy by inducing autophagy via inhibiting the AKT/mTOR pathway and increasing the ROS level in esophageal cancer cells 185. In addition, AIFCM such as ganoderma lucidum polysaccharide, curcumin and artesunate could improve the sensitivity of cancer cells to radiotherapy by regulating apoptosis-related proteins including survivin, Bcl2, and Bax. 186-188. For example, artesunate was reported to have a radiosensitizing effect in glioblastoma cells, which decreased the expression of survivin and increased the DNA damage response induced by ionizing irradiation 188. EGCG has been reported to exert a radiosensitization effect by inducing apoptosis via miR-34a/Sirt1/p53 signaling pathway in hepatoma cells H22, while exhibiting opposite radioprotective effects in normal hepatocyte cells 189.

**Figure 7:**
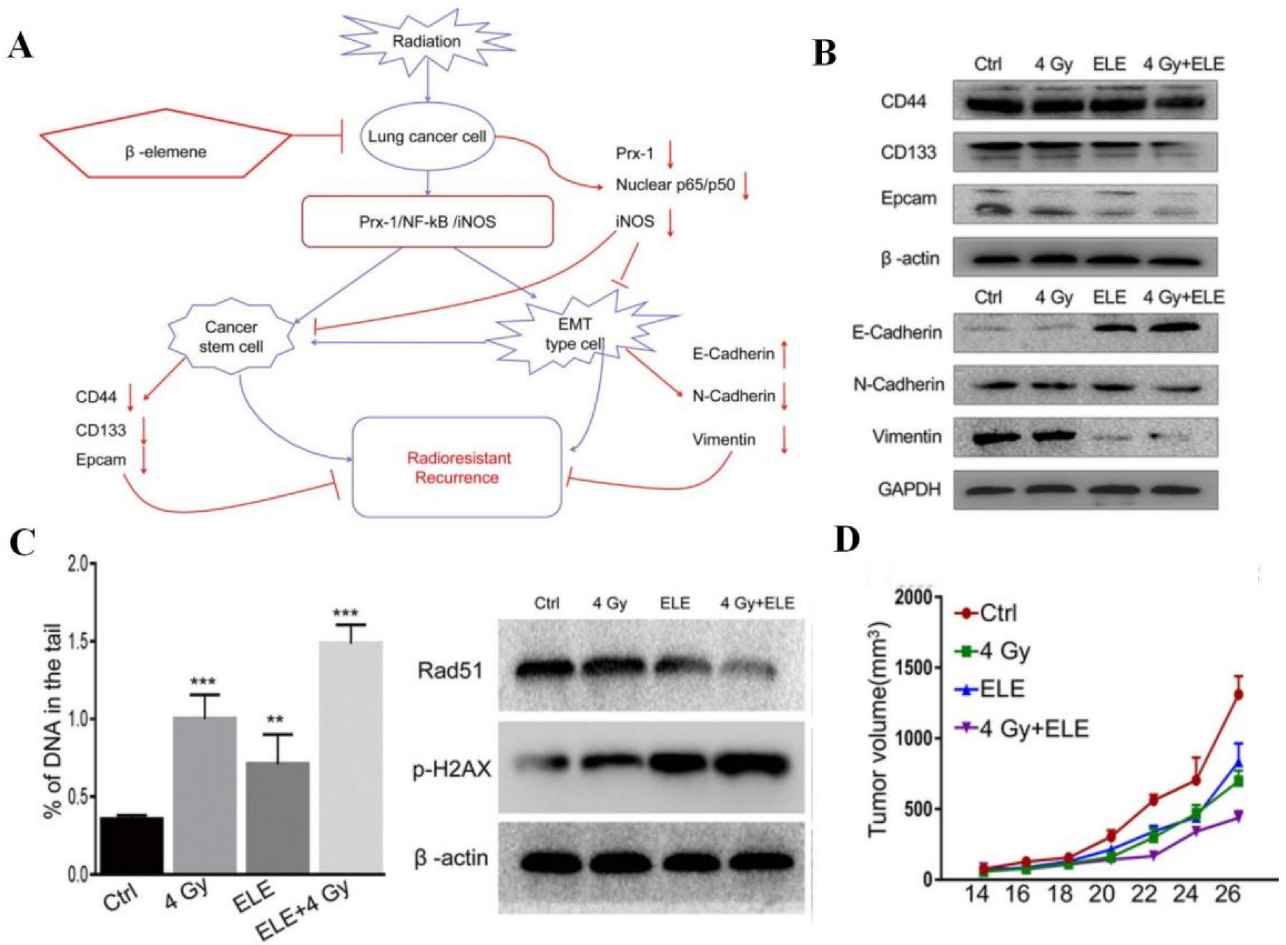
The combination of β-elemene and radiotherapy for radioresistant non-small-cell lung cancer treatment. (A) The illustration of β-elemene overcoming the radioresistance of A549 cells by suppressing the radiation-induced epithelial-mesenchymal transition (EMT) and cancer stem cells (CSCs) transdifferentiation via the Prx-1/NF-κB/iNOS pathway. (B) The downregulation of EMT makers (N-cadherin and vimentin) and CSC markers (CD133, CD44, and epcam) by β-elemene in radiotherapy. (C) The combination therapy induced DNA damage, increased γ-H2AX (double-strand break marker) expression and decreased Rad51 (double-strand break repair protein) expression in A549 cells. (D) The enhanced therapeutic effect of β-elemene combined with radiotherapy in A549 xenograft mouse model. Reproduced from 192 with permission from Zou et al.

Apart from facilitating tumor cell death, AIFCM can affect TME-related processes and factors, such as tumor hypoxia, CSCs, angiogenesis and inflammation, to improve the efficacy of radiotherapy. For example, berberine was found to reverse the radioresistance of esophageal squamous cancer by significantly inhibiting the expression of hypoxia-related HIF-1 and angiogenesis-associated VEGF, which contributed to the radiotherapy resistance and the alleviation of radiation damage 190. When combined with radiation for colon cancer treatment, honokiol, a polyphenol derived from the genus *Magnolia*, efficiently suppressed the Notch signaling pathway and its ligand Jagged-1 as well as target gene Hes-1 that mediated radiation resistance and CSCs promotion, to inhibit stemness and restore the effects of radiotherapy 191. Besides, by suppressing the Prx-1/NF-κB/iNOS pathway, β-elemene could downregulate EMT makers (N-cadherin and vimentin) and CSC markers (CD133, CD44, and epcam), inhibit the radiation-induced EMT and CSC, and thus promote the radiosensitivity of A549 cells in radiotherapy (Figure 7) 192. In addition, Wu's group reported that EGCG could attenuate the induction of angiogenesis by gamma radiation in breast cancer patients within the combination of EGCG and ionizing radiation 193. Moreover, Liang et al. used tetrandrine to reduce the inflammatory factor release of A549 tumor tissue to alleviate inflammation and improve the tumor inhibition effect in radiotherapy 194.

Radiotherapy plays a central role in current tumor treatment strategies. However, incomplete tumor eradication, radioresistance and the influence on the TME induced by radiation may result in tumor recurrence and metastasis. AIFCM have been found to promote radiosensitization and tumor inhibitory effect of radiotherapy by inducing autophagy, cell cycle arrest, DNA damage and apoptosis in cancer cells. Moreover, AIFCM could help to regulate the tumor microenvironment after radiotherapy, including inhibiting CSCs, and alleviating tumor hypoxia and inflammation, thus reversing the resistance of tumor cells to radiotherapy and promoting the therapeutic outcome. Additionally, some AIFCM especially those with antioxidant and ROS-scavenging ability have been reported to reduce the side effects of radiotherapy to normal tissue. For example, curcumin and resveratrol were found to prevent the radiotherapy-induced bone marrow toxicity and genotoxicity 195, 196. On the other hand, resveratrol could also act as a radiosensitizer by inducing ROS generation and apoptosis in cancer cells 197. Therefore, there are possibilities that the application of AIFCM with ROS regulation function in radiotherapy may have opposite effects on cellular ROS level and different effects on radiotherapy, which could be relevant to application dosage or cell type 198. It is necessary to carefully investigate the mechanisms of AIFCM in regulating cellular redox homeostasis in order to achieve the expected therapeutic outcome. In conclusion, the development of rational radiotherapy-AIFCM combinations could be beneficial to maximize therapeutic effects, elevate radiosensitization and combat intractable tumor metastasis.

### 2.5 AIFCM Combined with Immunotherapy

Immunotherapy, which exploits the host's immune system to inhibit cancer, is one of the most common modalities in cancer therapy 199. Recently, several significant advancements have been made in immunotherapy. Different types of cancer immunotherapies, such as antibodies, cytokines immune check point inhibitors and cancer vaccines, have been developed for inducing long-term tumor inhibition, which is a promising strategy for combating tumor metastasis 200, 201. However, tumors can develop multiple tolerance mechanisms to escape recognition and destruction by the immune system, such as releasing immunosuppressive molecules, preventing immune cell infiltration, and forming a tumor immunosuppressive microenvironment, etc 200. In recent years, Chinese medicine has shown unique and potent effects in regulating tumor microenvironment and human immunity. For examples, different Chinese medicine decoctions were found to inhibit the immune suppression effect of regulatory T cells (Treg), monocytic myeloid-derived suppressor cells (Mo-MDSC), and promote the proliferation of cytotoxic T cells for enhanced immunotherapy in cancer patients 202, 203. In specific, some AIFCM have been reported to increase the sensitivity of tumor cells to immunotherapeutic agents and facilitate tumor eradication by promoting the antibody-mediated cell death, the secretion of pro-inflammatory cytokines, the proliferation and activation of antitumor lymphocytes and immune checkpoint inhibition (Figure 3) 204, 205.

First, AIFCM can inhibit cancer in combination with antibodies that could trigger antibody-dependent cell-mediated cytotoxicity (ADCC) and contribute to the killing of antibody-recognized cells by immune cells 206. For examples, apigenin, which is a flavone that existed in celery and Chinese medicine *Rhizoma polygoni cuspidati*, was combined with epidermal growth factor receptor (EGFR) inhibitor cetuximab to synergistically down-regulate p-EGFR, p-Akt, p-STAT3 and Cyclin D1 in head and neck cancers. The combination therapy achieved more efficient tumor inhibition than single agent which could be attributed to the ADCC and apoptosis induced by cetuximab and apigenin, respectively 207. In addition, AIFCM could also be applied with antibodies against overexpressed receptors in cancer cells for combination therapy. For example, Ortiz et al. designed an antibody-avidin fusion protein for targeting and killing transferrin receptor (TfR)-overexpressed human malignant hematopoietic cells. The combination of the fusion protein and gambogic acid exerted potent cytotoxicity via TfR-dependent and TfR-independent pathways, respectively 208.

**Figure 8:**
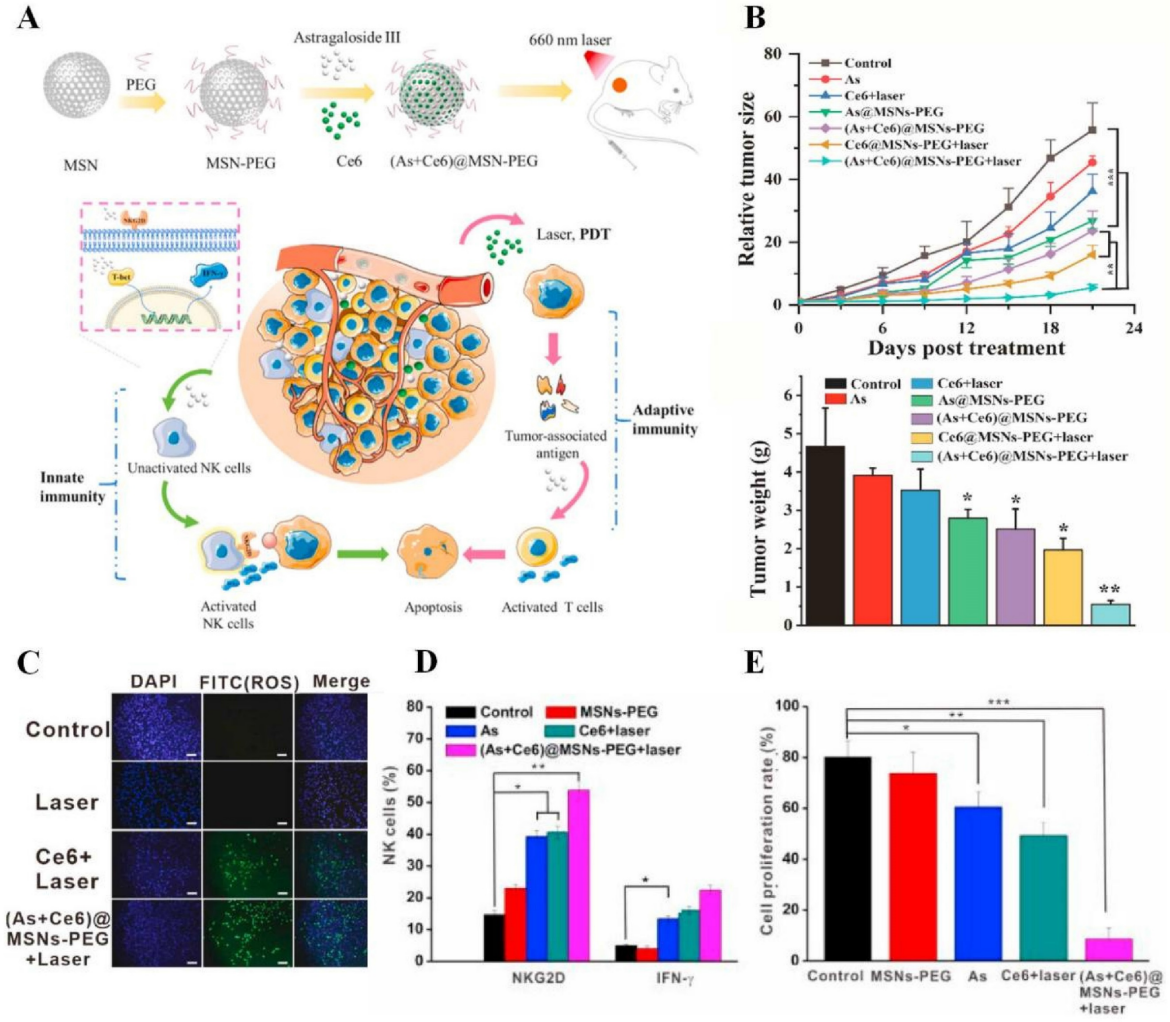
The combination immunotherapy based on astragaloside III and photodynamic therapy (PDT) for the treatment of colon cancer. (A) Schematic illustration of synergistic immunotherapy mediated by the astragaloside III activated NK cells and the PDT activated T cells. (B) The combination therapy significantly reduced the tumor size and weight of CT26-tumor bearing mice. (C) The combined treatment showed improved ROS generation effect in CT26 cells. The synergistic immunotherapy promoted (D) NK cell activation and (E) CT26 cell inhibition effect. Reproduced from 159 with permission from Elsevier.

Moreover, AIFCM have the ability to enhance cytokine-induced therapeutic effects and promote the activation of antitumor leukocytes. Cytokines are a range of proteins that regulate the immune system, and some cytokines can stimulate leukocytes to kill cancer cells 209. In a study by Guo's group, IL-2 and IL-15 were utilized to activate peripheral blood mononuclear cells (PBMC) to produce cytokine-induced killer cells (CIKs). Resveratrol was found to increase the sensitivity of human promyeloblastic leukemia to CIK-mediated cytolysis by enhancing the expression of natural-killer group 2D (NKG2D) ligands and activating the TRAIL pathway 210. Similarly, Zhao et al. combined paclitaxel and IL-2 for synergistic therapy against melanoma. Low-dose paclitaxel could remodel the immunosuppressive TME and increase tumor immunogenicity to facilitate the immune activation induced by IL-2. The combination of paclitaxel and IL-2 significantly inhibited tumor growth and the occurrence of metastasis in tumor-bearing mice 211. Besides, astragaloside III has been found to promote the activation of NK cells and the release of IFN-γ, which realized a synergistic therapeutic effect in combination with the PDT-induced adaptive immune response for the treatment of colon cancer (Figure 8) 159.

Additionally, AIFCM have been considered as potential candidates for enhancing the therapeutic effect of some major immunotherapeutic modalities in cancer treatment, including cancer vaccine therapy, and immune checkpoint blockade therapy. Among these immunotherapy modalities, cancer vaccine therapy is mainly designed to activate tumor-specific T lymphocytes to induce an active immune response against cancer 212. For example, AIFCM could help to prevent immune tolerance by inducing the maturation of Dendritic cells (DCs), which play central roles in vaccination owing to their capacity to capture, process, and present antigens to T cells 213. Chen et al. found that EGCG could promote DC maturation and thus enhance the anticancer immune response induced by a chimeric DNA vaccine composed of connective tissue growth factor and mesothelin, which is a tumor-associated antigen highly expressed in ovarian cancer, pancreatic cancer and malignant mesothelioma 214. Furthermore, considering that the immunosuppressive TME can suppress the therapeutic effect of vaccine therapy by regulating the interaction of tumor-infiltrating leukocytes, cytokines and inhibitory receptors, the modulation of TME by AIFCM could enhance cancer vaccine therapy 215. For instance, Hou et al. found that curcumin can modulate the immunosuppressive TME, as it significantly decreased the number of myeloid-derived suppressor cells (MDSCs) and regulatory T cells, and reduced levels of immunosuppressive factors as well as increased levels of pro-inflammatory cytokines by suppressing STAT3 pathways in combination with Trp2 vaccine, thus enhancing the Trp2 vaccine-induced cytotoxic T cell response and antitumor efficacy on melanoma 216.

On the other hand, immune checkpoint blockade (ICB) therapy is a promising immunotherapy that works by inhibiting the binding of immune checkpoint proteins, such as cytotoxic T lymphocyte antigen 4 (CTLA-4) and programmed cell death 1 protein (PD-1) on T cells to their ligands, in order to reverse the inactivation and exhaustion of T cell, and thus restore the function of T cells to kill tumor cells. Generally, the CTLA-4 overexpressed on Tregs and PD-L1 on tumor cells could bind to the immune checkpoint proteins on T cells to inhibit T cell activation, restrain antitumor immune response, and finally construct an immune suppressive environment 217, 218. Various AIFCM such as resveratrol, triptolide, curcumin and paclitaxel have been found to block immune checkpoints or regulate the tumor immune microenvironment for enhanced immunotherapeutic outcomes. For example, resveratrol could act as immune checkpoint inhibitor by suppressing the expression of PD-1 on T cells 219. While triptolide could down-regulate PD-L1 expression in cancer cells by inhibiting Jak2/STAT1 pathway, which helped to inhibit tumor growth and metastasis in cancer treatment 220. On the other hand, curcumin was shown to suppress CTLA-4 expression and the immunosuppressive cytokine (such as TGF-β and IL-10) production by Tregs, leading to the inhibition of immunosuppressive activity of Tregs 221. Moreover, curcumin could inhibit STAT3 in cancer cells and DCs for promoting the antitumor T cell responses, which reversed the immunosuppression in tumor microenvironment and showed a synergistic antitumor effect with the treatment of anti-PD-1/PD-L1 antibodies 222. In addition, Yang et al. found that paclitaxel could induce immunogenic cell death of tumor cells and promote antitumor immunity in mouse models. The combination therapy of paclitaxel and PD-1 antibody synergistically increased the infiltration and activation of T cells and DCs in tumor microenvironment, promoting the antitumor immune response of anti-PD-1 immunotherapy 223.

Immunotherapy represents an efficient and long-term anticancer approach. Moreover, it not only eradicates primary tumors but also inhibits metastatic tumors. Based on these advantages, immunotherapy is supposed to be the most promising approach for current cancer therapy 224. However, tumors develop multiple mechanisms to escape from the immune system, which limits the efficacy of immunotherapy 225. AIFCM help to facilitate the effects of immunotherapy by multiple mechanisms, such as inducing immunogenic cell death, modulating the TME, enhancing antigen presentation. In the combinational immunotherapy, AIFCM have complicated mechanisms for regulating tumor immune microenvironment, showing the features of multi-target and multi-pathway. For example, curcumin could not only inhibit the STAT3 pathway and immune checkpoint, but also suppress the immunosuppressive cytokine production and Treg activity. These multiple immunomodulatory effects made curcumin a promising adjuvant for cancer vaccine therapy and immune checkpoint blockade therapy 216, 221, 222. Therefore, AIFCM have shown great potential in improving the therapeutic effect of cancer immunotherapy. However, the targets and molecular mechanisms of AIFCM related to immunoregulation still need further investigation, as novel immunoregulatory pathways or targets have been found with the lacking of corresponding regulators. For instance, few studies on AIFCM acting as immune checkpoint inhibitors for CTLA-4 and especially those new checkpoint targets (such as Ang-2, NKG2A) have been reported 218. Thus, it would be fruitful to identify more active AIFCM with the ability to modulate the immune system and to develop more combination therapies of immunotherapeutic agents and AIFCM in future research.

## Clinical application of AIFCM in cancer combination therapy

Since long ago, Chinese medicine has been traditionally applied as dietary supplements or adjuvants for the treatment of cancer patients. Currently, the effects of AIFCM in clinical cancer treatment can be divided into the following parts: reducing toxic side effects, enhancing patient immunity and enhancing therapeutic effects.

The clinical effectiveness of AIFCM in PTT/PDT and gene therapy is less well studied, even when preclinical studies have shown the great potential of AIFCM in promoting the therapeutic effect of these cancer therapies. Several clinical trials have been carried out for evaluating the use of photosensitizer hypericin in PDT for treating cancer like skin cancer or mesothelioma 226, 227. However, numerous studies have shown that AIFCM achieved satisfactory clinical therapeutic results in combination therapies cooperated with chemotherapy, radiotherapy and immunotherapy. Among them, many kinds of active ingredients and composite formulas from Chinese medicine were found to prevent or relieve the toxic side effects induced by chemotherapy (such as nephrotoxicity, cardiotoxicity and hepatotoxicity, etc.), radiotherapy (such as pneumonitis, dermatitis and myelosuppression, etc.) and immunotherapy (such as immune-related adverse events) in patients 228-230. In addition, composite formulas from Chinese medicine are widely used as complementary therapies or dietary supplements to improve the immunity of patients in those clinical cancer treatments through a variety of mechanisms including regulating immune cells, and reducing inflammation 231-233.

On the other hand, various clinical trials have focused on the role of AIFCM in enhancing therapeutic outcomes in cancer patients through antitumor toxicity and therapeutic sensitization effect. For example, AIFCM such as silybin 234, genistein 235, 236 and curcumin 122 have been reported to improve the chemotherapeutic effect for cancer patients in clinical trials. In the combination therapy of regorafenib and silybin for treating metastatic colorectal cancer patients, silybin could increase the clinical efficacy of regorafenib and prevent the regorafenib-induced liver damage. The underlying mechanism may be related to the synergistic ROS promotion and apoptosis induction effect of silybin and regorafenib 234. In another phase I/II clinical study for the treatment of metastatic colorectal cancer, Genistein was found to improve the efficacy of chemotherapy when combined with FOLFOX (chemotherapy regimen using folinic acid, fluorouracil, and oxaliplatin) or FOLFOX-Bevacizumab. Herein, genistein has a sensitization effect on fluorouracil or platinum-based chemotherapy and can reduce tumor resistance to chemotherapy 235. It is worth pointing out that clinical studies of those AIFCM acting as drug resistance inhibitors in chemotherapy are still insufficient, and the corresponding large clinical trials need to be proposed 237, 238. A similar situation has been encountered in clinical studies of AIFCM as sensitizers for radiotherapy, with the majority of clinical trials of AIFCM (such as paclitaxel, curcumin and genistein) for sensitizing radiotherapy still ongoing, and a few studies demonstrating the effectiveness of AIFCM in improving the therapeutic outcomes of radiotherapy 193, 198, 239. For instance, fructus bruceae oil (oral administration, 20mL, 3 times daily for 12 weeks) was reported to reduce the side effect like esophageal obstruction and improve the therapeutic result of radiotherapy in patients with grade II-III squamous cell carcinoma 239. As for immunotherapy, AIFCM have shown a variety of abilities to enhance immunotherapy including recruiting lymphocytes, reducing immunosuppressive cells and increasing the immune-enhancing cytokines, etc. Many clinical trials on the use of AIFCM such as paclitaxel 240, 241, curcumin 242, 243 and lentinan 244 in immunotherapy like cancer vaccine therapy and immune checkpoint blockade therapy have been reported, and received enhanced therapeutic outcomes.

## 4. Conclusions

In recent years, AIFCM have been shown to improve the therapeutic effect and reduce toxic side effects of various anticancer modalities in combination therapy through different mechanisms, including targeting cell signaling pathways, regulating protein expression and cytokine secretion, and regulating the tumor microenvironment (Table 1). First, AIFCM can improve the efficacy of conventional chemotherapy and reduce its side effects by enhancing tumor suppression, reversing drug resistance, and overcoming metastasis. Second, the combined treatment of RNA or plasmid and AIFCM can silence the proto-oncogene and exert synergistic cytotoxic activity. Third, AIFCM combined with radiotherapy improves the sensitivity of cancer cells to radiotherapy and reduces radiation toxicity. Fourth, AIFCM and phototherapy drugs can synergistically achieve enhanced antitumor efficacy, and reduce therapy resistance, tumor recurrence and nonspecific damage to normal tissues. Fifth, some AIFCM can modulate the tumor immune microenvironment and enhance the immune response, thereby promoting the efficacy of immunotherapy. Overall, the application of AIFCM in tumor combination therapy exhibits multi-target and multi-channel properties, as well as the advantages of low toxicity as being natural compounds, obtaining more effective therapeutic results than monotherapy. Moreover, it's worth noting that some therapeutic modalities could promote the anticancer effectiveness of AIFCM not only via synergistic effect, but also by enhancing their cellular uptake or reversing the drug resistance (such as phototherapy and gene therapy) 137, 155.

Therefore, the AIFCM-containing combination therapy would be a promising strategy for effective cancer treatment. However, compared with the massive library of Chinese medicines, the AIFCM studied in anticancer combination therapy was just a drop in the bucket. Moreover, although AIFCM have been demonstrated to enhance treatment efficacy and reduce adverse reactions in clinical trials involving chemotherapy, radiotherapy and immunotherapy, the clinical effect of AIFCM in gene therapy and phototherapy remains understudied and needs further exploration. Thus, more efforts are demanded to discover new AIFCM with tumor inhibition activity and to develop new AIFCM-based combination therapies for clinical application. Moreover, the mechanisms by which AIFCM improve the antitumor efficacy are complex and involve many players ranging from cancer cells and the TME to the host physiology 245. In addition, the interactions of AIFCM and therapeutic agents that are applied in each cancer therapy may have a synergistic, antagonistic or even negative impact on the therapeutic effect. Therefore, considering that current researches mostly focus on the specific adjuvant effects of AIFCM in tumor therapy, it is necessary to study the functions of AIFCM in anticancer combinational therapy in a more comprehensive and detailed manner.

Furthermore, exquisite control of the combination drugs in the tumor tissue will be essential for optimum synergistic antitumor effects. Nano delivery systems offer an intelligent platform to co-deliver different therapeutic agents with precise ratios into tumor tissue and release them specifically in cancer cells 246. Several studies have demonstrated the satisfying outcome of using nanocarriers to co-administer AIFCM and other therapeutic agents for combination therapy 247, 248. Not only for disease treatment, some nanoparticles have also been used for molecular imaging, which enables AIFCM-loaded nanosystems to achieve better antitumor effects as synergistic theragnostic platforms 249, 250. In summary, AIFCM are excellent candidates for improving cancer therapies due to their diverse anticancer effects. The application of AIFCM in this regard is merely in its initial stage, and exciting progress can be expected in the near future.

## Figures and Tables

**Table 1:** Summary of the application of AIFCM in cancer combination therapy.

Combination therapy	Chinese medicine	Active ingredients	Specific mechanism	Reference
Chemotherapy	Curcuma longa	Curcumin	Inhibition of tumor proliferation; downregulation of BCRP, IAP family, Bcl-2, NF-κB, AP-1, integrin α_v_β_3_, c-FLIP; ROS promotion; reduction of the Smad2 phosphorylation and β-catenin level via TGF-β and PI3K/AKT signaling pathways, reverse EMT; inhibition of both non-CSCs and CSCs, mammosphere formation	27, 78, 85, 87, 93, 111, 120
Chemotherapy	Pueraria lobata, Vietnamese Sophora Root and Net Cliffbean	Genistein	Shifting the balance of Bax/Bcl-2 proteins, increasing the expression of caspase-3 and caspase-9	31
Chemotherapy	Scutellaria baicalensis	Baicalein	Increasing the expression of p53, inducing imbalance in Bax/Bcl-2, increasing levels of caspase-3, caspase-9 and PARP-1	33
Chemotherapy	Radix stephania tetrandrae	Tetrandrine	Increasing Bim and decreasing Mcl-1, promoting ROS production and caspase-induced apoptosis; suppressing P-gp activity; inactivating the WNT/β-catenin pathway, inhibiting EMT	35, 68, 112
Chemotherapy	Coptis chinensis	Berberine	Inducing G0/G1-phase blockade	40
Chemotherapy	Tripterygium wilfordii	Triptolide	Decreasing cyclin D1 and cyclin expression, inducing cell cycle arrest; anti-angiogenic effect mediated by VEGFR-2 inhibition	41, 118
Chemotherapy	Rhizoma zeodaria	β-elemene	Inducing cell cycle arrest, increasing CHK2 and reducing CDC2 activity	42
Chemotherapy	Taxus brevifolia	Paclitaxel	Promoting the polymerization of tubulin, blocking cell cycle at the G2/M phase	46
Chemotherapy	Catharanthus roseus	Vincristine	Disrupt tubulin polymerization, inducing cell cycle arrest at M phase	49
Chemotherapy	Camptotheca acuminata	Camptothecin	Topoisomerase I (TOP1) inhibitor, inhibiting DNA synthesis	51
Chemotherapy	Arsenolite	Arsenic trioxide	Inhibiting PARP-1 activity, preventing DNA damage repair	54
Chemotherapy	Sophora flavescens	Matrine	Suppressing PI3K/Akt/mTOR pathway, MRP1 and P-gp down-regulation, autophagy induction	69
Chemotherapy	Rhizoma polygoni cuspidati	Resveratrol	MRP1 and P-gp down-regulation	75
Chemotherapy	Corydalis yanhusuo	Glaucine	Binding to the substrate site of MRP1 and P-gp for competitive inhibition	77
Chemotherapy	Nelumbo nucifera, Nymphaea caerulea	Nuciferine	Inhibiting Nrf2 and HIF-1α via PI3K/AKT pathway, down-regulating the P-gp and BCRP levels	79
Chemotherapy	Andrographis paniculata	Andrographolide	Preventing Bax degradation, facilitating apoptosis; VEGF, VEGF-R2 and CD31 down-regulation	83, 116
Chemotherapy	Ginseng	Ginsenoside	Inhibiting hypoxia-induced EMT and stemness	91
Chemotherapy	Bupleurum	Quercetin	Decreasing the levels of IL‐8, IL‐6, and VEGF	96
Gene Therapy	Camellia sinensis	Epigallocatechin gallate	siRNA carriers	131
Gene Therapy	Garcinia hanburyi	Gambogic acid	InducING cell apoptosis	132
Gene Therapy	Curcuma longa	Curcumin	STAT3 inhibition, tumor growth suppression; gap junction proteins up-regulation, GJIC improvement	134 135
Gene Therapy	Rhizoma polygoni cuspidati	Resveratrol	Elevating the transactivation of p53 activity; up-regulating the gap junction proteins and improving GJIC	133 136
PTT	Toad Venom	Gamabufotalin	Inhibiting COX-2/PGE2 pathway, down-regulating the level of TNF-α and IL-6, and suppressing the PTT-induced inflammatory response	148
PTT	Bupleurum	Quercetin	HSP70 inhibitor	150
PTT	Garcinia hanburyi	Gambogic acid	HSP90 inhibitor	149
PDT	Curcuma longa	Curcumin	Antitumor cytotoxicity	162
PDT	Artemisia annua	Artemisinin	Promoting ROS generation, antitumor cytotoxicity	165
PTT/PDT	Taxus brevifolia	Paclitaxel	Antitumor cytotoxicity	145, 171
PTT/PDT	Camptotheca acuminata	Camptothecin	Antitumor cytotoxicity; reducing the hypoxia-responsive HIF-1α expression and normalizing the tumor vasculature, overcoming the PDT resistance; DNA topoisomerase-I inhibition	147, 153, 161, 163, 164, 167, 169
PTT/PDT	Artemisinin derivatives	Artesunate	Antitumor cytotoxicity	155, 170
Radiotherapy	Arsenolite	Arsenic trioxide	Inhibiting PI3K/AKT pathway and activating ERK1/2 pathway, inducing cell cycle arrest and autophagy	184
Radiotherapy	Garcinia hanburyi	Gambogic acid	Inhibiting the AKT/mTOR pathway and increasing ROS level, inducing autophagy	185
Radiotherapy	Artemisinin derivatives	Artesunate	Radiosensitization effect, decreasing survivin expression, increasing DNA damage response	188
Radiotherapy	Camellia sinensis	Epigallocatechin gallate	Radiosensitization effect, inducing apoptosis via miR-34a/Sirt1/p53 signaling pathway; attenuating angiogenesis	189, 193
Radiotherapy	Coptis chinensis	Berberine	Inhibiting the expression of HIF-1 and VEGF, reversing radioresistance	190
Radiotherapy	Magnolia	Honokiol	Suppressing the Notch signaling pathway, inhibiting radiation resistance and CSCs promotion	191
Radiotherapy	Rhizoma zeodaria	β-elemene	Radiosensitization effect, suppressing the Prx-1/NF-kB /iNOS pathway, inhibiting EMT and CSC	192
Radiotherapy	Radix stephania tetrandrae	Tetrandrine	Reducing the inflammatory factors, alleviating inflammation	194
Immunotherapy	Rhizoma polygoni cuspidati	Pigenin	Down-regulation of p-EGFR, p-Akt, p-STAT3 and Cyclin D1, promotion of antibody-mediated cytotoxicity and apoptosis	207
Immunotherapy	Garcinia hanburyi	Gambogic acid	Promoting antibody-mediated cytotoxicity	208
Immunotherapy	Rhizoma polygoni cuspidati	Resveratrol	Sensitizing CIK-mediated cytolysis by enhancing the expression of NKG2D ligands and activating the TRAIL pathway; suppressing the expression of PD-1	210, 219
Immunotherapy	Taxus brevifolia	Paclitaxel	Remodeling the immunosuppressive TME and increasing tumor immunogenicity; inducing immunogenic cell death, increasing the infiltration and activation of T cell and DCs	211, 223
Immunotherapy	Astragalus membranaceus	Astragaloside III	Promoting the activation of NK cells and the release of IFN-γ	159
Immunotherapy	Camellia sinensis	Epigallocatechin gallate	Promoting DC maturation, enhancing the anticancer immune response	214
Immunotherapy	Curcuma longa	Curcumin	Modulating the immunosuppressive TME, reducing MDSCs, Tregs and immunosuppressive factor levels, increasing levels of pro-inflammatory cytokines, suppressing STAT3 pathways; suppressing CTLA-4 expression, inhibiting Tregs activity	216, 221, 222
Immunotherapy	Tripterygium wilfordii	Triptolide	Down-regulating PD-L1 expression, inhibiting Jak2/STAT1 pathway	220
